# Long-term disparities and mediators of psychological distress in mothers with and without out-of-home care experience: analysis of prospective cohort data

**DOI:** 10.1136/bmjopen-2025-111174

**Published:** 2026-06-22

**Authors:** Jeongeun Park, Rachel M Hiller, Katherine Shelton, Bethan Carter, Eva A Sprecher, Erin Mckeaveney, Jeri L Damman, Charlotte Robinson, Tara Ramsay-Patel, Lisa Holmes

**Affiliations:** 1 School of Education and Social Work, University of Sussex, Brighton, UK; 2 Population, Policy and Practice Department, University College London Great Ormond Street Institute of Child Health, London, UK; 3 Division of Psychology & Language Sciences, University College London, London, London, UK; 4 School of Psychology, Cardiff University, Cardiff, UK; 5 School of Psychology, University of Ulster, Coleraine, UK

**Keywords:** Mothers, Postpartum Women, PSYCHIATRY, PUBLIC HEALTH, Psychological Stress, MENTAL HEALTH

## Abstract

**Abstract:**

**Objectives:**

Care-experienced individuals are at elevated risk of mental health difficulties, yet less is known about their mental health needs in motherhood. This study aims to explore (a) disparities in trajectories of psychological distress in care-experienced versus non-care-experienced mothers as they raise their child from age 3 to 14, and (b) putative postpartum-associated pathways shaping disparities in maternal distress at child age 14.

**Design and setting:**

We conducted longitudinal secondary data analysis using the prospective United Kingdom Millennium Cohort Study data.

**Participants:**

11 252 mothers and their children were included in the analysis, including mothers who experienced out-of-home care in their childhood (N=156).

**Outcomes:**

The primary outcomes were psychological distress of mothers at child ages 3, 5, 7, 11 and 14.

**Results:**

Relative to non-care-experienced mothers, care-experienced mothers were consistently more likely to experience distress across child ages 3–14 (coefficient (B)=1.36 (95% CI 0.85 to 1.87) *p*<0.001). The association between maternal care experience and increased maternal distress in their child’s adolescence was mediated by mental health difficulties (B=0.57 (95% CI 0.18 to 0.96) *p*=0.005), lower locus of control (B=0.31 (95% CI 0.11 to 0.51) *p*=0.002), lower income (B=0.28 (95% CI 0.15 to 0.41) *p*<0.001) and lower social support (B=0.16 (95% CI 0.05 to 0.28) *p*=0.006) in their child’s infancy.

**Conclusions:**

Our evidence underscores the potential value of holistically considering postpartum mental health, psychosocial and financial resources in maternal and early childhood public health interventions to disrupt the cumulative inequity likely faced by care-experienced mothers and prevent their mental ill-health in the long term.

**STRENGTHS AND LIMITATIONS OF THIS STUDY**

- This study is the first attempt to provide life course insights into long-term disparities in maternal psychological distress based on out-of-home care experience and putative postpartum pathways shaping distress disparities.
- Although our sample was representative of the British children born in 2000/2002, partly through oversampling ethnic minority and disadvantaged groups, representativeness cannot be assumed for mothers, and the care-experienced group is generally under-represented in population surveys.
- We used the presence of care experience for our research purpose; however, this measure can be limited to understanding maternal distress based on diverse care histories such as duration, type and stability of care experience.
- Future research should examine the role of detailed maternal social care, parent-level and child-level factors in shaping maternal distress and its consequences, potentially using administrative data to boost sample size and a mixed-methods study to understand the quality of healthcare experiences.

## Background

 Individuals with care experience in childhood have been found to be at risk of poor mental health and well-being. While being referred to by various terms in different contexts, such as local authority care in the UK, out-of-home care refers to children who are involved with child welfare services (hereafter, out-of-home care is referred to as care experience (We use the term ‘care experience’ to refer to out-of-home care experience, reflecting the view of a care-experienced live experience advisor, as this term is considered more inclusive and person-centred.). A decision has been made for these children to be placed in care, most commonly with foster carers. Care experience typically results from a history of adverse childhood experiences, with child abuse and neglect being the most common reasons for entering the care system in the UK.^1^,^3^ The substantial mental health and well-being needs of care-experienced young people and their persistence into adulthood are well-documented.^4^,^10^ However, less is known about these needs during motherhood. In this paper, we examine long-term disparities in maternal psychological distress between mothers with and without care experience as they raise their child through childhood and into adolescence. We also explore putative postpartum-related pathways that may shape maternal mental health disparities during their child’s adolescence.

Becoming a mother is a major life event, and motherhood is found to be a source of resilience for some care-experienced mothers.^11^ Nevertheless, motherhood, particularly the perinatal period, can be challenging for women’s mental health and well-being.^12^ Emerging evidence suggests that care-experienced mothers tend to face increased risks of experiencing psychological distress^13 14^ than non-care-experienced mothers within the first year after birth.^15 16^ While featuring depressive and anxiety symptoms,^17 18^ this maternal psychological distress has been shown to present a risk to women’s health^19^ as well as their children’s mental health and cognitive development in the general^20^,^22^ and care-experienced population.^23 24^

Yet, empirically, there has been limited focus on the psychological distress of care-experienced mothers beyond the first year after birth (In this study, we broadly use the term of postpartum referring to the first year after birth). Research shows that maternal distress can evolve, as parenthood demands different physical and mental investments and rewards for parents across their child’s developmental phases.^25 26^ Parents, particularly mothers, are found to become more prone to stress exposures when their children reach adolescence.^27 28^ Care-experienced mothers, especially younger women, might experience more vulnerability to distress than non-care-experienced mothers. Qualitative findings suggest that care-experienced mothers continued to navigate pressures from existing and additional mental health and systemic challenges from the pregnancy and parenting after birth.^29^,^31^ However, to date, it is unknown how disparities in distress trajectories unfold over time between mothers with and without care experience throughout their child’s childhood and adolescence. Additionally, it remains unclear about the potential postpartum-associated pathways through which maternal care experience shapes maternal psychological distress during their child’s adolescence. Childhood adversities do not inevitably translate into adult psychopathology.^32^ However, the life course perspective suggests that early adversities can lead to a chain of social disadvantages across life domains over time, which can, in turn, exacerbate mental health difficulties later in life.^33 34^ The maternal care experience in childhood is found to be associated with multiple disadvantages during the child’s infancy, including income deprivation, mental health difficulties, limited social support and a diminished sense of locus of control, as well as having a child with low birth weight.^15 16^ These factors have been, in turn, identified as predictors for worsening maternal psychological distress in the general population.^35^,^39^ Yet, empirical examination into this within care-experienced populations is lacking, and it is uncertain what roles these factors play in shaping the distress of care-experienced mothers during their child’s adolescence. Overall, addressing these knowledge gaps is crucial to informing modifiable targets to reduce maternal distress in the long-term through the universal and targeted maternal and early childhood public health intervention,^40 41^ especially for care-experienced mothers.

Our study addresses these gaps in the literature by using the UK’s longitudinal Millennium Cohort Study (MCS) data collected at child ages 9 months, 3, 5, 7, 11 and 14.^42^ We first examined how maternal distress trajectories vary between mothers with and without care experience, across child ages 3–14. We hypothesised that care-experienced mothers were more likely to experience distress than non-care-experienced mothers (hypothesis 1). We examined the patterns of maternal distress trajectories between these groups with no a priori assumptions due to the limited evidence base. Subsequently, we investigated whether the relationship between mothers’ childhood care experience and heightened maternal distress at child age 14 was mediated by the factors at 9 months after birth, including lower income, higher prior mental health difficulties, lower social support, lower locus of control and lower child birth weight (hypothesis 2).

## Method

### Data and sample

We used the data from the UK’s MCS,^42^ which is an ongoing birth cohort study, following the lives of a nationally representative sample of British children born in 2000/2002 across the four UK nations. The MCS oversampled children from ethnically minoritised and socioeconomically disadvantaged groups.^43^ The analytic sample for this study was created in two steps. First, out of the original sample at sweep 1 (N=18 552), we selected birth mothers who provided consistent information about their relationship to the MCS cohort in the sweeps in which they participated (N=18 465). We then further restricted the sample to those who completed the sweep 6 survey to apply a survey weight (N=11 252). Participants in this final analytic sample largely resided in England (64.13%), followed by Wales (14.81%), Scotland (11.20%) and Northern Ireland (9.87%) at sweep 1. This distribution closely mirrored that of the original sample, which was designed to be broadly representative of the total UK population at baseline through sampling techniques and weighting.^43 44^ Out of 11 252 mothers, 156 (1.4%) were reported to have had care experience in childhood. Of those care-experienced mothers, 60% (N=92) had experienced family-based care and 40% (N=64) had experienced group-based care. Most care-experienced mothers had spent less than 2 years in care (41%; n=64); 24% (n=38) were in care from 2 to 5 years; and 33% (n=52) had spent over 5 years in care. Two care-experienced mothers did not report the length of time in the care system.

### Measures

#### Outcome variable: maternal psychological distress

Self-reported maternal psychological distress (MPD) was measured using the validated Kessler 6 scale,^45^ collected when their MCS child was aged 3, 5, 7, 11 and 14 years old. This scale is designed to screen for serious mental illness by asking how often individuals experience symptoms of psychological distress during the last 30 days. Items concern how often individuals feel depressed, hopeless, restless/fidgety, that everything is an effort, worthless and nervous. Individuals responded to each item, using a 6-point Likert scale (1=all of the time, 5=none of the time, 6=can’t say). The ‘c*an’t say*’ response was treated as a missing value. For analysis purposes, the original values were reverse-coded on a scale from 0 to 4, producing a total range of 0 to 24 and higher values indicate higher distress. We used the scores as a continuous variable to enable the comparison of raw distress scores over time across groups of mothers. In line with the previous MCS studies focused on populations with social disadvantages,^22 35^ we also used the following cut-offs: normal (0–5) and distress (6-24), as a reference point to understand the pattern of clinically elevated psychological distress. All distress measures at each sweep showed good internal consistency reliability (Cronbach’s α: sweep 2 (0.86), sweep 3 (0.87), sweep 4 (0.88), sweep 5 (0.89), Sweep 6 (0.88)).

#### Predictor: maternal care experience in childhood

Maternal care experience in childhood was derived from questions asking whether participants had spent any time living away from both parents before age 17. If yes, a follow-up question was made to participants to indicate where they mainly lived before age 17, including children’s home, foster parents, boarding school, living with relatives, prison/young offenders institute/Borstal or some other place. Mothers who had lived in a care placement, including foster care and children’s home, were classified as having care experience (0=no care-experience, 1=care-experience).

### Mediators

Mediators included the continuous measure of the self-reported MCS child’s birth weight (kilos) as well as the following maternal factors collected when the MCS child was 9 months old.

#### Household income

Self-reported weekly Organisation for Economic Co-operation and Development equivalised household income (£, Great British Pound) was used,^42^ which adjusts for the composition and size of the household. Higher values indicate higher income.

#### Prior maternal mental health

Scores from the modified Rutter Malaise Scale^13 46^ were used to indicate the prior maternal mental health at 9 months postpartum. This scale includes nine binary items (1=yes, 0=no) about an individual experience of the following symptoms: (a) ‘feel tired most of the time*’*, (b) ‘often feel miserable or depressed*’*, (c) ‘often get worried about things*’*, (d) ‘often get into a violent rage*’*, (e) ‘often suddenly become scared for no good reason*’*, (f) ‘easily upset or irritated*’*, (g) ‘constantly keyed up or jittery*’*, (h) ‘every little thing gets on your nerves and wear you out*’*, (i) ‘heart often races like mad*’* (Cronbach’s α=0.72). A total score was used by summing scores from the nine items. Higher scores indicate higher mental health difficulties, with a range of 0–9.

#### Maternal perceived social support

Perceived social support was originally measured using the three items on a 6-point scale (strongly agree (1) to strongly disagree (5) and can’t say (6)), including (a) *I have no one to share feelings with,* (b) *I have other parents to talk to and* (c) *family would help if I had financial problems*. ‘Can’t say’ response was treated as missing. (b) and (c) statements were reverse-coded, so that higher values indicate higher perceived support. The summed scores were used for analysis (Cronbach’s α= 0.52), resulting in a total range of 3–15.

#### Maternal locus of control

Locus of control was largely defined as one’s perceived control over one’s life and was originally measured on a 3-point scale by asking (a) whether respondents get what they want out of life (b) whether they have control over life and (c) whether they run life the way they want (see the online supplemental document for the full response categories). *‘Can’t say’ was treated as missing. Reverse coding was applied to (b) and (c) statements, and summed scores (range of 3–6) were used to indicate that higher values mean feeling more in control* (Cronbach’s α= 0.62).

#### Controls

Maternal age and maternal ethnicity (white and minoritised ethnic groups) were included. The earliest available information from sweep 2 on maternal grandmother’s and maternal grandfather’s work status when the mother was 14-year old was used as a proxy measure of maternal grandparents’ socioeconomic status.

### Statistical analysis

Missing values were present in the outcomes, mediators and controls, with an average missingness of approximately 10% (see online supplemental table 1 for detailed information). Missingness was assumed to be missing at random and addressed using multiple imputation. Details of the imputation procedures are provided later in this section. To examine the trajectories of maternal distress between mothers with and without care experience across child ages 3–14 years, we used a piecewise random slope model.^47^ This statistical decision was driven by the initial descriptive exploration of the maternal psychological distress, whereby their average scores did not seem to change before age 7 and began to increase after age 7 among mothers, regardless of care experience (see table 1). This was also supported by the tenability of introducing a two-piece linear function of time. Hence, we fitted two linear splines with a knot point of age 7 to separately capture a pattern of change before and after age 7. We statistically tested interaction terms between this time and care experience variables to examine whether the trajectories of maternal distress pre-age and post-age 7 differed by mothers’ care experience status. Subsequently, to examine the effects of multiple mediators in the relationship between maternal care experience and maternal distress at child age 14, we conducted a parallel mediation analysis.^48^

**Table 1:** Maternal psychological distress at each child’s age

	Non-care-experienced mothers (N=11 096)	Care-experienced mothers (N=156)	P value
Maternal psychological distress at child age 3			
Valid N	8345	100	
Mean (SD)	3.17 (3.68)	5.56 (4.98)	p<0.001
(Min, median, max)	(0, 2, 24)	(0, 4, 19)	
Normal group N (%)	6798 (81.46)	56 (56)	
Distress group N (%)	1547 (18.54)	44 (44)	
Missing N (%)	2751 (24.79)	56 (35.90)	
Maternal psychological distress at child age 5			
Valid N	9436	126	
Mean (SD)	3.05 (3.73)	4.87 (5.38)	p<0.001
(Min, median, max)	(0, 2, 24)	(0, 3, 24)	
Normal group N (%)	7742 (82.05)	86 (68.25)	
Distress group N (%)	1694 (17.95)	40 (31.75)	
Missing N (%)	1660 (14.96)	30 (19.23)	
Maternal psychological distress at child age 7			
Valid N	9234	111	
Mean (SD)	3.02 (3.78)	4.99 (5.31)	p<0.001
(Min, median, max)	(0, 2, 24)	(0, 3, 24)	
Normal group N (%)	7566 (81.94)	68 (61.26)	
Distress group N (%)	1668 (18.06)	43 (38.74)	
Missing N (%)	1862 (16.78)	45 (28.85)	
Maternal psychological distress at child age 11			
Valid N	9457	119	
Mean (SD)	3.88 (4.27)	6.82 (6.13)	p<0.001
(Min, median, max)	(0, 3, 24)	(0, 5, 24)	
Normal group N (%)	7075 (74.81)	62 (52.10)	
Distress group N (%)	2382 (25.19)	57 (47.90)	
Missing N (%)	1639 (14.77)	37 (23.72)	
Maternal psychological distress at child age 14			
Valid N	9835	123	
Mean (SD)	4.31 (4.18)	7.03 (5.83)	p<0.001
(Min, median, max)	(0, 3, 24)	(0, 6, 24)	
Normal group N (%)	6992 (71.09)	58 (47.15)	
Distress group N (%)	2843 (28.91)	65 (52.85)	
Missing N (%)	1261 (11.36)	33 (21.15)	

Note: Unweighted counts are reported. Mann-Whitney U test samples were conducted to test the relationship between continuous and categorical variables.

While the overall sample size of this study was large, the care-experienced population size is small, and the care experience variable had imbalanced group cell sizes (see table 2). Despite ongoing debate, a two-level linear random slope model was found to be robust with unequal and small group sizes (<10) in producing unbiased estimates of fixed effects and their SEs (which is the focus of this study), compared with using a single-level linear model.^49^ Regarding the parallel mediation model, maximum likelihood estimation with robust (Huber-White) SEs and a scaled test statistic that is asymptotically equal to the Yuan-Bentler test statistic^50^ was used. To handle the missingness, we performed two separate multiple imputations, so that the imputation models are aligned with the statistical models. Each statistical model requires a different data structure derived from the same underlying data. The piecewise random slope model uses the repeated maternal distress data from child age 3–14, whereas the parallel mediation analysis is based on the cross-sectional maternal distress data at child age 14. For the piecewise random slope model, multilevel multiple imputation was performed by generating 10 imputed datasets and considering the two-level hierarchical structure in the outcome data. A single-level multiple imputation with 10 imputed datasets was conducted for the parallel mediation model. 10 imputed datasets were generated based on Rubin’s rules^51^ to estimate the uncertainty introduced by missing data, given the overall level of 10% missingness in our data. See online supplemental table 1 for the comparison of descriptive statistics between the original and imputed datasets. The survey weight was used to account for the attrition and sampling bias in the MCS data. All analyses were conducted in R programming, using mice, lme4, lavaan and lavaan.mi packages. We used the Strengthening the Reporting of Observational Studies in Epidemiology (STROBE) reporting guideline and checklist to form this manuscript.^52^

**Table 2:** Baseline sample characteristics (at child age 9 months)

	Non-care-experienced mothers (N=11 096)	Care-experienced mothers (N=156)	P value
Maternal age			
Valid N	11 094	156	
Mean (SD)	28.95 (5.78)	25.29 (5.69)	
(Min, max)	(14, 51)	(15, 39)	p<0.001
Missing N (%)	2 (0.02)	0 (0)	
Maternal ethnicity			
Valid N	11 076	156	
White group N (%)	9299 (83.80)	140 (89.74)	
Minoritised ethnic groups N (%)	1777 (16.01)	16 (10.26)	p=0.05
Missing N (%)	20 (0.18)	0 (0)	
Maternal grandmother’s work status			
Valid N	9721	102	
Did not work N (%)	3406 (30.7)	50 (32.05)	
Worked N (%)	6315 (56.91)	52 (33.33)	p=0.005
Missing N (%)	1375 (12.39)	54 (34.62)	
Maternal grandfather’s work status			
Valid N	9139	92	
Did not work N (%)	725 (6.53)	19 (12.18)	
Worked N (%)	8414 (75.83)	73 (46.79)	p<0.001
Missing N (%)	1957 (17.64)	64 (41.03)	
Household income at child age 9 months			
Valid N	10 988	155	
Mean (SD)	312.03 (203.71)	195.75 (149.12)	
(Min, median, max)	(14.45, 270.24, 1282.77)	(25.63, 157.37, 961.60)	p<0.001
Missing N (%)	108 (0.97)	1 (0.64)	
Prior mental health at child age 9 months			
Valid N	10 680	150	
Mean (SD)	1.66 (1.74)	2.73 (2.22)	
(Min, max)	(0, 9)	(0, 9)	p<0.001
Missing N (%)	416 (3.75)	6 (3.85)	
Social support at child age 9 months			
Valid N	10 195	136	
Mean (SD)	12.47 (2.06)	11.04 (2.59)	
(Min, max)	(3, 15)	(3, 15)	p<0.001
Missing N (%)	901 (8.12)	20 (12.82)	
Locus of control at child age 9 months			
Valid N	8286	100	
Mean (SD)	5.76 (0.62)	5.36 (0.96)	
(Min, max)	(3, 6)	(3, 6)	p<0.001
Missing N (%)	2810 (25.32)	56 (35.90)	
MCS child birth weight			
Valid N	10 951	155	
Mean (SD)	3.37 (0.58)	3.22 (0.60)	
(Min, max)	(0.39, 7.23)	(0.94, 4.76)	p=0.001
Missing N (%)	145 (1.31)	1 (0.64)	

Unweighted counts are reported. Mann-Whitney U test samples were conducted to test the relationship between continuous and categorical covariates. To test the association between categorical variables, Fisher’s exact test was used.

MCS, Millennium Cohort Study.

### Results

Table 2 shows maternal characteristics stratified by care experience at baseline (when the child was 9 months old) based on the observed data. See online supplemental table 1 for the descriptive profiles based on the imputed datasets. Care-experienced mothers were slightly younger than non-care-experienced mothers at baseline. Bivariate correlation results indicated that repeated maternal distress scores were highly correlated with each other over time (online supplemental table 2). Maternal care experience in childhood was associated with the outcomes and mediators in the expected manner: having care experience in mothers’ childhood was associated with having lower income, higher prior mental health difficulties, lower social support, lower locus of control and lower childbirth weight at 9 months postpartum. Additionally, maternal care experience was correlated with elevated distress at child ages 3, 5, 7, 11 and 14 (table 1, online supplemental table 3). These mediators were associated with higher distress scores at child age 14 (online supplemental table 2).

### Trajectories of maternal psychological distress by care experience

**Figure 1:**
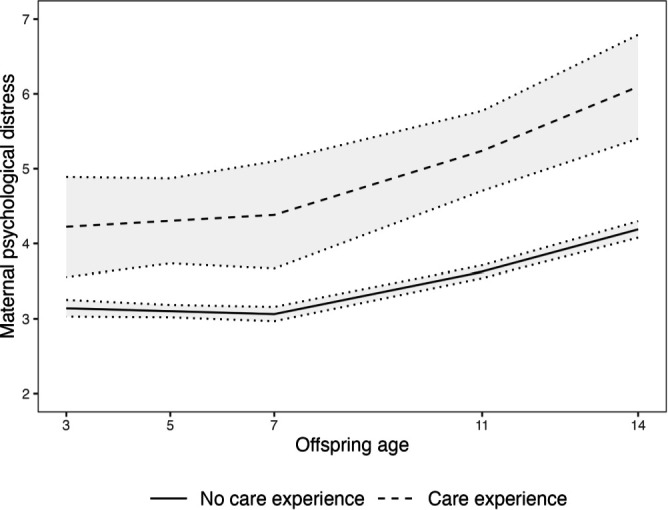
Predicted levels of maternal psychological distress by care experience across their child’s childhood and adolescence. Results from model 1 were plotted. Pooled adjusted predicted values of maternal psychological distress were plotted while adjusting for maternal age, maternal ethnicity, maternal grandmother’s work status, and maternal grandfather’s work status using the ggpredict function from the ggeffects R package. 95% CIs were plotted in shade. The survey weight was used.

Table 3 presents results from a series of piecewise random slope models. Non-significant interaction terms ($B_{Time1xcareexperience};B_{Time2xcareexperience})$) were removed iteratively to select a more parsimonious model (online supplemental table 4). The final results from model 3 suggest that maternal distress, on average, did not significantly change before child age 7 ($B_{Time1}$ = −0.04, 95% CI (−0.10 to 0.03), p=0.247). As shown in figure 1, mothers’ distress levels, on average, tended to increase after child age 7, regardless of care experience ($B_{Time2}$=0.57, 95% CI (0.51 to 0.63), p<0.001). In line with the hypothesis 1, the average distress level of care-experienced mothers was higher than that of non-care-experienced mothers ($B_{careexperience}$=1.36, 95% CI (0.85 to 1.87), p<0.001). The magnitude of this difference did not significantly vary over time across the child’s developmental phases, as substantiated by the tenability of removing the interaction terms, as mentioned above.

**Table 3:** Results from the piecewise random slope models

Term	Model 1		Model 2		Model 3	
	Estimate	(95% CI)	Estimate	(95% CI)	Estimate	(95% CI)
Fixed effects						
(Intercept)	4.98***	(4.67 to 5.29)	4.98***	(4.67 to 5.29)	4.98***	(4.67 to 5.29)
Time 1	−0.04	(−0.10 to 0.03)	−0.04	(−0.10 to 0.03)	−0.04	(−0.10 to 0.03)
Time 2	0.56***	(0.50 to 0.62)	0.56***	(0.50 to 0.62)	0.57***	(0.51 to 0.63)
Care experience	1.32*	(0.59 to 2.04)	1.20***	(0.61 to 1.78)	1.36***	(0.85 to 1.87)
Time1×Care Experience	0.12	(−0.29 to 0.53)				
Time2×Care Experience	0.29	(−0.19 to 0.78)	0.36	(−0.05 to 0.77)		
Maternal age	−0.07***	(−0.08 to −0.06)	−0.07***	(−0.08 to −0.06)	−0.07***	(−0.08 to −0.06)
Maternal ethnicity	1.09***	(0.89 to 1.28)	1.09***	(0.89 to 1.28)	1.09***	(0.89 to 1.28)
Maternal grandmother’s work status	0.15*	(0.02 to 0.29)	0.15*	(0.02 to 0.29)	0.15*	(0.02 to 0.29)
Maternal grandfather’s work status	0.63***	(0.38 to 0.87)	0.63***	(0.38 to 0.87)	0.63***	(0.38 to 0.87)
Random effects						
Intercept variance	8.95		8.95		8.95	
Time 1 variance	1.82		1.82		1.82	
Time 2 variance	2.31		2.31		2.31	
Covariance between intercept and time 1	2.14		2.14		2.14	
Covariance between intercept and time 2	−1.68		−1.68		−1.68	
Covariance between time1 and time 2	−0.91		−0.91		−0.91	
Residual	7.86		7.86		7.86	
N	56 260		56 260		56 260	
N (ID)	11 252		11 252		11 252	
ICC	0.53		0.53		0.53	

Time 1 indicates a rate of change in maternal distress from child age 3 to 7. Time 2 indicates a rate of change in maternal distress from child age 7 to 14. Ethnicity (reference category=white); maternal grandmother’s work status (reference category=employed); maternal grandfather’s work status (reference category=employed). Pooled estimates and uncertainty are reported for 10 imputed datasets. Variances and covariances were not identical across Models, and they appeared so due to rounding the values to two decimal places. The survey weight was used. P value was calculated using Satterthwaite’s method.

*p<0.05, ***p<0.001.

ICC, Intraclass Correlation Coefficient.

### The role of mediators in the association between maternal care experience and maternal psychological distress at the child’s age of 14

**Figure 2:**
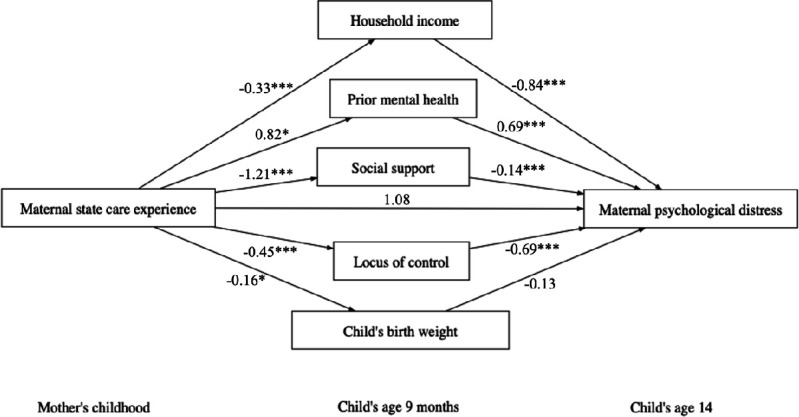
Visualisation of mediation effects. Results from the parallel mediation model were reported (n=11 252). Pooled unstandardised effects were reported due to the imbalanced subgroup sizes of mothers. Please see 95% CI for each estimate in table 3. The survey weight was used. See the standardised estimates reported in the online supplemental table 6. *p<0.05, ***p<0.001.

Table 4 shows key results from the parallel mediation model. See online supplemental tables 5 and 6 for the model fit results and full unstandardised and standardised estimates. Partially confirming hypothesis 2, maternal care experience did not directly predict maternal distress at child age 14 once all the postpartum-associated mediators were included in the model ($B_{Care\;experience\to MPD14}$=1.08, 95% CI (−0.44 to 2.61), p=0.163). As shown in figure 2, part of the effect of maternal care experience on maternal distress at child age 14 was mediated through the postpartum factors. Mothers with care experience were more likely to experience greater mental health difficulties ($B_{Care\;experiene\to Prior\;mental\;health\to MPD14}$=0.57, 95% CI (0.18 to 0.96), p=0.005), lower locus of control ($B_{Care\;experience\to Locus\;of\;control\to MPD14}$=0.31, 95% CI (0.11 to 0.51), p=0.002), lower household income ($B_{Care\;experience\to Household\;Income\to MPD14}=0.28$, 95% CI (0.15 to 0.41), p<0.001), lower social support ($B_{Care\;experience\to Social\;Support\to MPD14}$=0.16, 95% CI (0.05 to 0.28), p=0.006) at 9 months postpartum, which, in turn, predicted higher maternal distress at child age 14. Prior maternal mental health accounted for the largest part of the total effect of care experience on maternal distress (24%), followed by locus of control (13%), household income (12%) and social support (7%).

**Table 4:** Key results from the parallel mediation model

	Unstandardised estimate	95% CI	Proportion mediated
Care experience → MPD14	1.08	−0.44 to 2.61	
Household income → MPD14	−0.84***	−1.05 to −0.63	
Prior mental health → MPD14	0.69***	0.61 to 0.78	
Social support → MPD14	−0.14***	−0.21 to −0.06	
Locus of control → MPD14	−0.69***	−0.94 to −0.44	
Child’s birth weight → MPD14	−0.13	−0.34 to 0.08	
Care experience → Household income	−0.33***	−0.46 to −0.21	
Care experience → Prior mental health	0.82*	0.27 to 1.37	
Care experience → Social support	−1.21***	−1.82 to −0.6	
Care experience → Locus of control	−0.45***	−0.69 to −0.21	
Care experience → Child’s birth weight	−0.16*	−0.28 to −0.04	
Indirect effect			
Care experience → Household income → MPD14	0.28***	0.15 to 0.41	0.12
Care experience → Prior mental health → MPD14	0.57*	0.18 to 0.96	0.24
Care experience → Social support → MPD14	0.16*	0.05 to 0.28	0.07
Care experience → Locus of control → MPD14	0.31*	0.11 to 0.51	0.13
Care experience → Child birth weight → MPD14	0.02	−0.01 to 0.05	0.01
Total effect	2.43*	0.83 to 4.02	
Covariance			
Household income ↔ Prior mental health	−0.14***	−0.17 to −0.11	
Household income ↔ Social support	0.28***	0.24 to 0.32	
Household income ↔ Locus of control	0.09***	0.08 to 0.1	
Household income ↔ Child birth weight	0.02***	0.01 to 0.03	
Prior mental health ↔ Social support	−1.1***	−1.23 to −0.96	
Prior mental health ↔ Locus of control	−0.59***	−0.65 to −0.53	
Prior mental health ↔ Child birth weight	−0.04*	−0.07 to −0.02	
Social support ↔ Locus of control	0.5***	0.43 to 0.57	
Social support ↔ Child birth weight	0.06*	0.02 to 0.09	
Locus of control ↔ Child birth weight	0.02*	0.01 to 0.03	

N=11 252. Unstandardised effects are reported due to imbalanced group sizes of care experience. See the standardised estimates in the online supplemental table 6. Pooled results for 10 imputed datasets based on Rubin’s rules are reported. Household income was log-transformed. The controls, including maternal age, maternal ethnicity, maternal grandmother’s and maternal grandfather’s work status, were adjusted for.

* *p<0.05, **p<0.001.

MPD14, maternal psychological distress at child age 14.

## Discussion

Using the large-scale longitudinal data, we explored disparities in the trajectories of psychological distress between mothers with and without care experience across child ages 3–14, and potential postpartum-associated pathways shaping disparities at child age 14. Relative to non-care-experienced mothers, our evidence shows the experience of greater distress among care-experienced mothers while raising their children into childhood and adolescence. Increased vulnerability to distress was shown among both mother groups as their child entered adolescence. This might be influenced by shifts in developmental and environmental demands (eg, school transition) and parent–child relationship dynamics,^27 28^ although a careful interpretation was needed as this coincided with the Great Recession in the UK.^53^ We demonstrate the potential cumulative inequity faced by care-experienced mothers, where their motherhood journey tended to be characterised by higher rates of mental health difficulties, lower financial means, lower locus of control and lower social support at 9 months postpartum, relative to non-care-experienced mothers. Our findings show that these factors partially contribute to mothers’ higher distress levels to varying degrees at child age 14, highlighting not only the need for psychological support (where indicated) but also the systemic inequities that these mothers can face.

Our findings uniquely demonstrate that poorer mental health at 9 months after birth was the strongest mediator of the relationship between maternal care experience and higher distress levels at child age 14. This finding underscores the importance of continuous mental health support, especially during motherhood. In the UK, amid facing greater likelihood of adverse mental health conditions while in care and after leaving care^5^,^10^ and barriers to receive sufficient support in transition to adulthood and/or adult mental health services,^11 29 54^ care-experienced mothers may be likely to face unique challenges, such as fear of or experience of traumatic child removal,^30^ and/or the stigma and bias associated with care experience and teen pregnancy.^55^,^57^ While navigating pre-existing mental health difficulties, this may create barriers in accessing and seeking support from the health services, which may exacerbate their distress over time.

Our results further highlight the simultaneous roles of additional structural and psychosocial resources during the child’s infancy in shaping maternal distress in the child’s adolescence. Extending the existing evidence,^11 15 16 29 31^ our study shows that income-based financial hardship at 9 months postpartum, which is more likely to be experienced by care-experienced mothers, predicted higher maternal distress at the child’s age of 14. Care-experienced individuals are likely to experience educational and social disadvantages during the compulsory schooling period,^58^,^62^ which can shape adult socioeconomic status.^63^ Additionally, they are legally required to move to independent living at a much younger age than the general population. This can create accelerated transitions to independent living^64^ and parenthood^57^ without necessarily familial support and the provision of suitable accommodation.^29^ This pressure may make it particularly challenging for care-experienced women to prepare to establish socioeconomic standing in adulthood and take a toll on their mental health during motherhood, as similarly shown with socioeconomic vulnerabilities.^35^

Our evidence supports the potential role of agency in shaping the mental health of care-experienced individuals,^65^ showing that care-experienced mothers’ lower locus of control at 9 months postpartum partly contributes to their higher maternal distress at the child’s age of 14. While further research is needed to understand the determinants of lower locus of control in care-experienced mothers, the feeling of powerlessness over their lives may partly originate from experiencing a childhood in a bureaucratised care system operated by adult professionals^66^ and/or perceived scrutiny of social and health services about their parenting capacity due to their care history.^67^ Meanwhile, contrary to our hypothesis, the mediating effect of social support in the relationship between care experience and maternal distress at child age 14 was significant but smaller, relative to those of other mediators. This might be because the social support measure used in this study may be insufficient to meaningfully capture the unique supports received by care-experienced mothers. Nevertheless, our evidence points to lower social support within 1 year of birth as one of the pathways to higher distress in care-experienced mothers at child age 14. Previous qualitative research suggests that care-experienced mothers require concerted support to reduce social isolation and build strong social connections, as relationships with birth families or formal carers can be complex and not necessarily guarantee emotional, financial or practical support^67^,^69^

### Strengths, limitations and future directions

To our knowledge, this study is the first of its kind to quantitatively examine long-term patterns of maternal psychological distress by care experience from a life course perspective, using large-scale data in the UK. A life course examination of care-experienced mothers’ mental health has been almost absent from the discourse. We provide new evidence on the timing of elevated distress while raising their children and postpartum maternal factors that likely contribute to elevated distress at the child’s age of 14 among care-experienced mothers. Despite the strengths, our study is not without limitations. While showing the social demographic characteristics consistent with the previous MCS research,^15 16^ the national representativeness of the MCS data was ensured for the MCS cohort child only, not for care-experienced mothers. Future research is encouraged to use (linked) administrative or nationally representative cohort data, potentially with mixed methods approaches, to boost the sample size of care-experienced mothers, as well as to explore diverse factors in shaping their psychological distress. Our study used the presence of care experience as the predictor to establish whether care experience in childhood can have a long-term impact on maternal distress over time. Nevertheless, given the heterogeneity in care experience,^70^,^72^ it would be beneficial to consider the role of detailed care histories (eg, placement types, duration, stability, age at entry) in maternal distress. Furthermore, antecedents and consequences of heightened maternal distress among care-experienced mothers can be examined by considering maternal characteristics (eg, early educational and health histories, single parenthood, the number of children and the intersection of ethnicity and care experience^73^), other health outcomes (eg, premature mortality)^74 75^ and child’s outcomes. Additionally, our social support and locus of control measures showed low reliability, and therefore, it is important to replicate the analysis with alternate measures. It would also be beneficial to examine the role of other child and parent-level mediators beyond the postpartum period. While this was beyond the scope of the present study, some variance in the maternal distress outcome remains to be explained, warranting further investigation, such as the role of parenting stress and coping mechanisms,^76 77^ child mental health,^78^ child–mother relationships^78^ or mother–partner relationships^79^ in the association between care experience and later distress. As explained in the Statistical analysis section, we used the two separate multiple imputation models, each specified to be congenial with its corresponding statistical model, to obtain valid statistical inference. Nevertheless, this approach generated different imputed values for the same missing observation, although the overall distributions of these values were similar (see online supplemental table 1). Additionally, the estimates from each statistical model were not directly comparable, as different sets of imputed datasets were used. Meanwhile, future research is advised to use better quality measures to control for the care-experienced mothers’ socioeconomic status in childhood, given the relatively high missingness in their grandparents’ work status measures.

### Public health implications

Our evidence of a persistent vulnerability to psychological distress among care-experienced mothers supports the current UK government’s efforts to promote maternal mental health through universal and targeted maternal and early childhood public health interventions, especially in the presence of adverse childhood experiences.^40 41^ For instance, the Family Nurse Partnership Programme in the UK^41 80^ currently supports first-time young mothers with a history of care experience and their families for the first 2 years of a child’s life. Our evidence of care-experienced mothers’ greater mental health needs reinforces the focus of this intervention on this group. However, further evidence is needed to understand its effectiveness in supporting the complex health needs of care-experienced mothers beyond the perinatal period, including building trusting and non-stigmatising relationships with healthcare professionals within the resource-constrained public healthcare settings.^81 82^ Our mediation evidence suggests that attention can be paid to reducing the cumulative inequity faced by care-experienced mothers, by providing holistic and preventative support ranging from maternal mental health to financial and psychosocial resources. While governmental provisions to support care leavers are gaining more attention in the UK,^83^ our evidence supports the need for strengthening more holistic support for care-experienced mothers, to avoid income deprivation and enhance locus of control and support networks, which can be associated with lower psychological distress during their child’s teen years.

## Conclusions

This UK-based study shows persistent vulnerability to psychological distress among care-experienced mothers, particularly in their child’s adolescence, compared with mothers with no care experience. Care experience was associated with maternal disadvantages at 9 months after birth across multiple life domains, including lower income, poorer mental health, lower locus of control and lower social support. These factors, in turn, were associated with higher maternal distress when their child was 14 years old. This highlights the potential importance of preventative and holistic maternal mental health and psychosocial support to disrupt social disadvantages based on care experience and enhance maternal mental health in the long term.

## Supplementary material

10.1136/bmjopen-2025-111174 online supplemental file 1

## Data Availability

Data are available in a public, open access repository.
