# Supplementary material for: Long-term disparities and mediators of psychological distress in mothers with and without out-of-home care experience: analysis of prospective cohort data
Source: BMJ Open. 2026 Jun 22;16(6):e111174. doi: 10.1136/bmjopen-2025-111174 (PMC13289143; doi:10.1136/bmjopen-2025-111174)
Supplement: online supplemental file 1 [file bmjopen-16-6-s001.docx]

**Title: Long-term Disparities and Mediators of Psychological Distress in Mothers with and without Out-of-Home Care Experience: Analysis of Prospective Cohort Data**

**Supplementary document**

1. **Locus of control variable**

Locus of control variable is measured using the three items: (a) whether respondents get what they want out of life (1=I never really seem to get what I want and 2=I usually get what I want out of life) (b) whether they have control over life (1=I usually have a free choice and control over my life and 2=Whatever I do has no real effect on what happens to me) and (c) whether they run life the way they want (1=Usually I can run my life more or less as I want to and 2=I usually find life’s problems just too much for me).

1. **Comparison of descriptive statistics between the original and imputed datasets**

**Supplementary Table 1 Comparison of descriptive statistics between the original and imputed datasets**

|  | **Original N** | **Imputed N** | **Missing (%)** | **Full sample** | | **Non-care-experienced mothers** | | **Care-experienced mothers** | |
| --- | --- | --- | --- | --- | --- | --- | --- | --- | --- |
|  |  |  |  | **Observed statistic** | **Imputed statistic** | **Observed statistic** | **Imputed statistic** | **Observed statistic** | **Imputed statistic** |
| **Multilevel multiple imputation for the piecewise random slope model (N=56260)**^1^ | | | | | | | | | |
| MPD at child age 3 | 8445 | 2807 | 25 | M=3.20  (SD=3.71) | M=3.41  (SE=0.05) | M=3.17  (SD=3.68) | M=3.38  (SE=0.05) | M=5.56  (SD=4.98) | M=4.88  (SE=0.44) |
| MPD at child age 5 | 9562 | 1690 | 15 | M=3.07  (SD=3.76) | M=3.23  (SE=0.04) | M=3.05  (SD=3.73) | M=3.21  (SE=0.04) | M=4.87  (SD=5.38) | M=4.6  (SE=0.45) |
| MPD at child age 7 | 9345 | 1907 | 17 | M=3.04  (SD=3.8) | M=3.22  (SE=0.05) | M=3.02  (SD=3.78) | M=3.20  (SE=0.05) | M=4.99  (SD=5.31) | M=4.64  (SE=0.43) |
| MPD at child age 11 | 9576 | 1676 | 15 | M=3.92  (SD=4.31) | M=3.95  (SE=0.04) | M=3.88  (SD=4.27) | M=3.92  (SE=0.04) | M=6.82  (SD=6.13) | M=6.03  (SE=0.51) |
| MPD at child age 14 | 9958 | 1294 | 12 | M=4.34  (SD=4.21) | M=4.33  (SE=0.04) | M=4.31  (SD=4.18) | M=4.30  (SE=0.04) | M=7.03  (SD=5.83) | M=6.28  (SE=0.48) |
| Maternal age | 56250 | 10 | 0 | M=28.90 (SD=5.79) | M=28.90  (SE=0.02) | M=28.95  (SD=5.78) | M=28.95  (SE=0.02) | M=25.29  (SD=5.67) | M=25.29  (SE=0.2) |
| Maternal ethnicity* | 56160 | 100 | 0 |  |  |  |  |  |  |
| Non-White |  |  |  | 16% | 16%  (SE=0.002) | 16.04% | 16%  (SE=0.002) | 10.26% | 10%  (SE=0.011) |
| Maternal grandmother’s work status* | 49115 | 7145 | 13 |  |  |  |  |  |  |
| Yes (%) |  |  |  | 64.82% | 64.20%  (SE=0.008) | 64.96% | 64%  (SE=0.007) | 50.98% | 57%  (SE=0.041) |
| Maternal grandfather’s work status* | 46155 | 10105 | 18 |  |  |  |  |  |  |
| Yes (%) |  |  |  | 91.94% | 91.60%  (SE=0.003) | 92.07% | 92%  (SE=0.003) | 79.35% | 84%  (SE=0.021) |
| **Single-level multiple imputation for the parallel mediation model (N=11252)** | | | | | | | | | |
| MPD at child age 14 | 9958 | 1294 | 12 | M=4.34 (SD=4.21) | M=4.44  (SE=0.04) | M=4.31  (SD=4.18) | M=4.40  (SE=0.04) | M=7.03  (SD=5.83) | M=7.02  (SE=0.48) |
| Maternal age | 11250 | 2 | 0 | M=28.90 (SD=5.79) | M=28.90  (SE=0.05) | M=28.95  (SD=5.78) | M=28.95  (SE=0.05) | 25.29  (SD=5.69) | M=25.29  (SE=0.46) |
| Log household income | 11143 | 109 | 1 | M=5.52  (SD=0.68) | M=5.52  (SE=0.01) | M=5.53 (SD=0.68) | M=5.53  (SE=0.01) | M=5.08  (SD=0.60) | M=5.08  (SE=0.05) |
| Prior mental health | 10830 | 422 | 4 | M=1.67  (SD=1.75) | M=1.68  (SE=0.02) | M=1.66  (SD=1.74) | M=1.67  (SE=0.02) | M=2.73  (SD=2.22) | M=2.73  (SE=0.18) |
| Social support | 10331 | 921 | 8 | M=12.45 (SD=2.08) | M=12.40  (SE=0.02) | M=12.47  (SD=2.06) | M=12.24  (SE=0.02) | M=11.04  (SD=2.59) | M=11.09  (SE=0.21) |
| Locus of control | 8386 | 2866 | 25 | M=5.76  (SD=0.63) | M=5.70  (SE=0.01) | M=5.76  (SD=0.62) | M=5.71  (SE=0.01) | M=5.36  (SD=0.96) | M=5.29  (SE=0.09) |
| Child birth weight | 11106 | 146 | 1 | M=3.37  (SD=0.58) | M=3.37  (SE=0.01) | M=3.37  (SD=0.58) | M=3.37  (SE=0.01) | M=3.22  (SD=0.60) | M=3.22  (SE=0.05) |
| Maternal ethnicity* | 11232 | 20 | 0 |  |  |  |  |  |  |
| Non-White |  |  |  | 15.96% | 16%  (SE=0.003) | 16.04% | 16%  (SE=0.003) | 10.26% | 10%  (SE=0.024) |
| Maternal grandmother’s work status* | 9823 | 1429 | 13 |  |  |  |  |  |  |
| Yes |  |  |  | 64.82% | 64%  (SE=0.005) | 64.96% | 64%  (SE=0.005) | 50.98% | 55%  (SE=0.049) |
| Maternal grandfather’s work status* | 9231 | 2021 | 18 |  |  |  |  |  |  |
| Yes |  |  |  | 91.94% | 91.40%  (SE=0.003) | 92.07% | 92%  (SE=0.003) | 79.35% | 82%  (SE=0.039) |

**Note:** MPD=Maternal psychological distress.

^1^ The same underlying data were used to conduct multiple imputations. For the multilevel multiple imputation, the length of the original dataset increased fivefold (N=56260) by converting it to the longitudinal format with time-varying repeated psychological distress measures.

* Missing values were excluded from percentage calculations.

1. **Bivariate relationships between the studied variables**

**Supplementary Table 2 A correlation matrix between care experience, mediators, and outcomes**

|  | **1** | **2** | **3** | **4** | **5** | **7** | **8** | **9** | **10** | **11** | **12** | **13** | **14** | **15** |
| --- | --- | --- | --- | --- | --- | --- | --- | --- | --- | --- | --- | --- | --- | --- |
| 1.MPD3 | 1 |  |  |  |  |  |  |  |  |  |  |  |  |  |
| 2.MPD5 | 0.58 | 1 |  |  |  |  |  |  |  |  |  |  |  |  |
| 3.MPD7 | 0.55 | 0.60 | 1 |  |  |  |  |  |  |  |  |  |  |  |
| 4.MPD11 | 0.51 | 0.51 | 0.57 | 1 |  |  |  |  |  |  |  |  |  |  |
| 5.MPD14 | 0.48 | 0.48 | 0.52 | 0.61 | 1 |  |  |  |  |  |  |  |  |  |
| 7.HI | -0.20 | -0.19 | -0.19 | -0.22 | -0.20 | 1 |  |  |  |  |  |  |  |  |
| 8.PMH | 0.48 | 0.42 | 0.42 | 0.38 | 0.38 | -0.15 | 1 |  |  |  |  |  |  |  |
| 9.SS | -0.26 | -0.24 | -0.22 | -0.25 | -0.22 | 0.24 | -0.31 | 1 |  |  |  |  |  |  |
| 10.LoC | -0.34 | -0.33 | -0.31 | -0.3 | -0.28 | 0.24 | -0.45 | 0.32 | 1 |  |  |  |  |  |
| 11.BW | -0.06 | -0.05 | -0.05 | -0.05 | -0.05 | 0.10 | -0.05 | 0.05 | 0.08 | 1 |  |  |  |  |
| 12.Age | -0.12 | -0.09 | -0.09 | -0.11 | -0.12 | 0.37 | -0.09 | 0.09 | 0.15 | 0.09 | 1 |  |  |  |
| 13.Ethnicity | 0.11 | 0.09 | 0.08 | 0.11 | 0.04 | -0.2 | 0.06 | -0.14 | -0.11 | -0.17 | -0.05 | 1 |  |  |
| 14.GME | -0.05 | -0.04 | -0.03 | -0.05 | -0.04 | 0.12 | -0.03 | 0.07 | 0.06 | 0.03 | 0.04 | -0.29 | 1 |  |
| 15.GPE | -0.09 | -0.09 | -0.08 | -0.09 | -0.08 | 0.16 | -0.09 | 0.09 | 0.09 | 0.04 | 0.14 | -0.08 | 0.16 | 1 |

**Note:** MPD3=Maternal psychological distress at child age 3; MPD5=Maternal psychological distress at child age 5; MPD7=Maternal psychological distress at child age 7; MPD11=Maternal psychological distress at child age 11; MPD14=Maternal psychological distress at child age 14; HI=Household income; PMH=Prior mental health; SS=Social support; LoC=Locus of control; BW=Child’s birth weight; GME=Maternal grandmother’s work status (2=Yes); GPE=Maternal grandfather’s work status (2=Yes). Ethnicity (2=Minoritised group). All Spearman correlation estimates were statistically significant at a 5% level. Unweighted results are reported.

**Supplemental Table 3 Correlations between care experience, outcomes and mediators and adjusted effect sizes**

| **Care experience** | **Correlation** | **Effect size** |
| --- | --- | --- |
| MPD3 | 0.07 | 0.60 |
| MPD5 | 0.06 | 0.47 |
| MPD7 | 0.06 | 0.48 |
| MPD11 | 0.08 | 0.65 |
| MPD14 | 0.07 | 0.61 |
| Household income | -0.07 | 0.57 |
| Prior mental health | 0.07 | 0.62 |
| Social support | -0.08 | 0.67 |
| Locus of Control | -0.07 | 0.60 |
| Child’s birth weight | -0.03 | 0.26 |
| Maternal age | -0.07 | 0.63 |
| Maternal grandmother's work status | -0.03 | n.a |
| Maternal grandfather's work status | 0.05 | n.a |
| Maternal ethnicity | -0.02 | n.a |

**Note:** MPD3=Maternal psychological distress at child age 3; MPD5=Maternal psychological distress at child age 5; MPD7=Maternal psychological distress at child age 7; MPD11=Maternal psychological distress at child age 11; MPD14=Maternal psychological distress at child age 14. Point-biserial correlation and adjusted effect size for unequal group sizes (See McGrath & Meyer (2006)) were calculated for the bivariate relationship between care experience, outcomes, and mediators. These point-biserial correlations were statistically significant at a 5% level. Phi coefficient was calculated for the bivariate relationship between care experience and categorical factors, including maternal grandmother's work status, maternal grandfather's work status, and ethnicity. Unweighted results are reported.

1. **Model comparison results from the piecewise linear random slope models**

**Supplementary Table 4 Model comparison results**

| **Statistic** | **Df1** | **Df2** | **Complete-data degrees of freedom** | **p.value** | **Relative increase in variance due to nonresponse** |
| --- | --- | --- | --- | --- | --- |
| Remove Time1 x care experience vs Model 1 | | | | | |
| 0.33 | 1 | 41.57 | 56243 | 0.569 | 0.45 |
| Remove Time1 x care experience; Time2 x care experience vs Model 1 | | | | | |
| 1.81 | 2 | 95.36 | 56243 | 0.169 | 0.57 |

**Note:** The multivariate Wald test was used to compare the two nested piecewise random slope models from the imputed datasets.

1. **Model fit results from the parallel mediation model**

**Supplementary Table 5 Parallel mediation model fits**

| **Model** | **Chisq** | **Df** | **p-value** | **Robust CFI** | **Robust TLI** | **SRMR** | **Robust RMSEA**  **[90% CI]** |
| --- | --- | --- | --- | --- | --- | --- | --- |
| Model | 0.932 | 2.000 | 0.627 | 1.000 | 1.010 | 0.011 | 0.000  [0.000, 0.017] |

**Note:** This model was based on the imputed datasets. Test statistics were pooled using the D4 pooling method.

1. **Unstandardised and standardised results from the parallel mediation model**

**Supplementary Table 6 Unstandardised and standardised results from the parallel mediation model**

|  | **Unstandardised estimate** | | **Standardised estimate** | |
| --- | --- | --- | --- | --- |
|  | **Estimate** | **95% CI** | **Estimate** | **95% CI** |
| Care experience → MPD14 | 1.08 | [-0.44,2.61] | 0.04 | [-0.01,0.09] |
| Household income → MPD14 | -0.84*** | [-1.05,-0.63] | -0.13*** | [-0.16,-0.1] |
| Prior mental health → MPD14 | 0.69*** | [0.61,0.78] | 0.28*** | [0.24,0.31] |
| Social support → MPD14 | -0.14*** | [-0.21,-0.06] | -0.06*** | [-0.1,-0.03] |
| Locus of control → MPD14 | -0.69*** | [-0.94,-0.44] | -0.11*** | [-0.15,-0.07] |
| Child’s birth weight → MPD14 | -0.13 | [-0.34,0.08] | -0.02 | [-0.04,0.01] |
| Maternal age → MPD14 | -0.01 | [-0.03,0.01] | -0.01 | [-0.04,0.02] |
| Maternal ethnicity → MPD14 | -0.52* | [-0.89,-0.14] | -0.04* | [-0.07,-0.01] |
| Maternal grandfather’s work status → MPD14 | 0 | [0,0] | 0 | [0,0] |
| Maternal grandmother’s work status → MPD14 | 0 | [0,0] | 0 | [0,0] |
| Care experience → Household income | -0.33*** | [-0.46,-0.21] | -0.07*** | [-0.1,-0.05] |
| Maternal age → Household income | 0.04*** | [0.04,0.05] | 0.39*** | [0.36,0.41] |
| Maternal ethnicity → Household income | -0.35*** | [-0.4,-0.29] | -0.18*** | [-0.2,-0.15] |
| Maternal grandfather’s work status → Household income | -0.27*** | [-0.32,-0.21] | -0.12*** | [-0.14,-0.09] |
| Maternal grandmother’s work status → Household income | 0.07*** | [0.03,0.1] | 0.05*** | [0.02,0.07] |
| Care experience → Prior mental health | 0.82* | [0.27,1.37] | 0.07* | [0.02,0.12] |
| Maternal age → Prior mental health | -0.02*** | [-0.03,-0.02] | -0.08*** | [-0.11,-0.05] |
| Maternal ethnicity → Prior mental health | 0.29* | [0.13,0.45] | 0.06*** | [0.02,0.09] |
| Maternal grandfather’s work status → Prior mental health | 0.33* | [0.12,0.53] | 0.05* | [0.02,0.09] |
| Maternal grandmother’s work status → Prior mental health | -0.03 | [-0.14,0.08] | -0.01 | [-0.04,0.02] |
| Care experience → Social support | -1.21*** | [-1.82,-0.6] | -0.09*** | [-0.13,-0.04] |
| Maternal age → Social support | 0.03*** | [0.01,0.04] | 0.07*** | [0.04,0.1] |
| Maternal ethnicity → Social support | -0.81*** | [-1.02,-0.6] | -0.13*** | [-0.16,-0.1] |
| Maternal grandfather’s work status → Social support | -0.44*** | [-0.67,-0.21] | -0.06*** | [-0.09,-0.03] |
| Maternal grandmother’s work status → Social support | 0.15* | [0.01,0.29] | 0.03* | [0,0.06] |
| Care experience → Locus of control | -0.45*** | [-0.69,-0.21] | -0.09*** | [-0.14,-0.04] |
| Maternal age → Locus of control | 0.01*** | [0.01,0.02] | 0.12*** | [0.09,0.15] |
| Maternal ethnicity → Locus of control | -0.21*** | [-0.28,-0.13] | -0.1*** | [-0.13,-0.06] |
| Maternal grandfather’s work status → Locus of control | -0.12* | [-0.21,-0.03] | -0.05* | [-0.08,-0.01] |
| Maternal grandmother’s work status → Locus of control | 0.04 | [-0.01,0.09] | 0.03 | [-0.01,0.06] |
| Care experience → Child’s birth weight | -0.16* | [-0.28,-0.04] | -0.04* | [-0.07,-0.01] |
| Maternal age → Child’s birth weight | 0.01*** | [0,0.01] | 0.08*** | [0.05,0.11] |
| Maternal ethnicity → Child’s birth weight | -0.22*** | [-0.27,-0.17] | -0.13*** | [-0.16,-0.1] |
| Maternal grandfather’s work status → Child’s birth weight | -0.04 | [-0.1,0.02] | -0.02 | [-0.05,0.01] |
| Maternal grandmother’s work status → Child’s birth weight | -0.02 | [-0.05,0.02] | -0.01 | [-0.04,0.02] |
| **Indirect effect** |  |  |  |  |
| Care experience → Household income → MPD14 | 0.28*** | [0.15,0.41] | 0.01*** | [0.01,0.01] |
| Care experience → Prior mental health → MPD14 | 0.57* | [0.18,0.96] | 0.02* | [0.01,0.03] |
| Care experience → Social support → MPD14 | 0.16* | [0.05,0.28] | 0.01* | [0,0.01] |
| Care experience → Locus of control → MPD14 | 0.31* | [0.11,0.51] | 0.01* | [0,0.02] |
| Care experience → Child birth weight → MPD14 | 0.02 | [-0.01,0.05] | 0 | [0,0] |
| **Total effect** | 2.43* | [0.83,4.02] | 0.08* | [0.03,0.14] |
| **Covariance** |  |  |  |  |
| Household income ↔ Prior mental health | -0.14*** | [-0.17,-0.11] | -0.13*** | [-0.16,-0.1] |
| Household income ↔ Social support | 0.28*** | [0.24,0.32] | 0.22*** | [0.19,0.25] |
| Household income ↔ Locus of control | 0.09*** | [0.08,0.1] | 0.21*** | [0.18,0.23] |
| Household income ↔ Child birth weight | 0.02*** | [0.01,0.03] | 0.06*** | [0.03,0.08] |
| Prior mental health ↔ Social support | -1.1*** | [-1.23,-0.96] | -0.29*** | [-0.32,-0.26] |
| Prior mental health ↔ Locus of control | -0.59*** | [-0.65,-0.53] | -0.46*** | [-0.49,-0.43] |
| Prior mental health ↔ Child birth weight | -0.04* | [-0.07,-0.02] | -0.04* | [-0.07,-0.02] |
| Social support ↔ Locus of control | 0.5*** | [0.43,0.57] | 0.32*** | [0.29,0.36] |
| Social support ↔ Child birth weight | 0.06* | [0.02,0.09] | 0.05* | [0.02,0.07] |
| Locus of control ↔ Child birth weight | 0.02* | [0.01,0.03] | 0.05* | [0.02,0.08] |
| **Variance** |  |  |  |  |
| MPD14 | 16.51*** | [15.54,17.49] | 0.81*** | [0.79,0.84] |
| Household income | 0.36*** | [0.35,0.38] | 0.77*** | [0.75,0.79] |
| Prior mental health | 3.17*** | [3.01,3.33] | 0.98*** | [0.97,0.99] |
| Social support | 4.47*** | [4.22,4.72] | 0.96*** | [0.95,0.97] |
| Locus of control | 0.52*** | [0.49,0.56] | 0.96*** | [0.94,0.97] |
| Child’s birth weight | 0.33*** | [0.31,0.35] | 0.97*** | [0.97,0.98] |

**Note:** N=11252. MPD14=Maternal psychological distress at child age 14. The unstandardised and standardised estimates for Figure 2 and Table 3 in the main manuscript are reported. Pooled results for 10 imputed datasets based on Rubin’s rules are reported. Please note that we fixed the non-significant direct path from maternal grandfather’s work status to MPD14 and from maternal grandmother’s work status to MPD14 to 0 to resolve a saturated model.

**References**

McGrath RE, Meyer GJ. When effect sizes disagree: the case of r and d. Psychological methods. 2006 Dec;11(4):386.
