# Supplementary material for: Long-term disparities and mediators of psychological distress in mothers with and without out-of-home care experience: analysis of prospective cohort data
Source: BMJ Open. 2026 Jun 22;16(6):e111174. doi: 10.1136/bmjopen-2025-111174 (PMC13289143; doi:10.1136/bmjopen-2025-111174)
Supplement: Supplementary file 2 [file bmjopen-2025-111174.reviewer_comments.pdf]

PEER REVIEW HISTORY

BMJ Open publishes all reviews undertaken for accepted manuscripts. Reviewers are asked to complete a checklist review form and are provided with free text boxes to elaborate on their assessment. These free text comments are reproduced below.

ARTICLE DETAILS

Title (Provisional)

Long-term Disparities and Mediators of Psychological Distress in Mothers with and without Out-of-Home Care Experience: Analysis of Prospective Cohort Data

Authors

Park, Jeongeun; Hiller, Rachel M; Shelton, Katherine; Carter, Bethan; Sprecher, Eva A; Mckeaveney, Erin; Damman, Jeri L; Robinson, Charlotte; Ramsay-Patel, Tara; Holmes, Lisa

---

VERSION 1 - REVIEW

**Reviewer**	**1**
**Name**	**Parsons, Sam**
**Affiliation**	**UCL IOE, Centre for Longitudinal Studies**
**Date**	**18-Nov-2025**
**COI**	**None**

Long-term Disparities and Mediators of Psychological Distress in Mothers with and without Out-of-Home Care Experience: Analysis of Prospective Cohort Data

I was very pleased to have the opportunity to review this manuscript. It makes use of an incredibly rich longitudinal data recourse, that includes a measure of maternal out-of-home care experience.

I have recommended a major revision, but feel in reality this lies somewhere between minor and major.

Abstract

I would suggest amending the ‘outcomes’ section to jjst include the outcomes – not the mediators.

‘into their child’s development’. Should this be ‘over their child’s development’?

Strengths and limitations

Representativeness cannot be assumed for mothers. This is correct, but maybe add that this 'representativeness' will almost certainly particularly apply to the care-experienced sample of mothers. Many of the mothers initially contacted would potentially be more likely to not take part if they have out-of-home care experience.

(and in background)

'care experience' 'social care' 'out-of-home-care' are all used. It would help the reader if one is used. I suggest out-of-home care experience or OHC

Page 4: Ref 12. Please check that child abuse and neglect are still the main reasons children go into care in the UK? This reference is quite old (2013) and I think there are increasingly older and unaccompanied (migrant) children in the care system.

Into childhood and adolescence. Amend to 'through childhood and into adolescence'?

Include 'measure' after distress and include what the measure is and reference it?

Malaise Inventory

Rutter, M., Tizard. J., & Whitmore, K. (1970). Education, health and behaviour. London: Longman.

Rodgers, B., Pickles, A., Power, C., Collishaw, S. & Maughan, B. (1999). Validity of the Malaise Inventory in general population samples. Social Psychiatry and Psychiatric Epidemiology, 34(6), 333-341. https://doi.org/10.1007/s001270050153

Page 5: 'as parenthood places' change to 'as parenthood demands'?

Page 6: find a better word for 'inquiries'

I am unconvinced about the inclusion of a child's birthweight as a mediator of psychological distress.

Methods

Page 7: the n=19243 is incorrect for the age 9 month sample. This is n=18,818 children and n=18,552 families.

I am unclear about the following sentence and resulting sample size which is then restricted to those who participated at age 14. This needs to be made much clearer to me.

'Out of the original sample at Sweep 1 (N=19,243), birth mothers who provided

consistent information about their relationship to the MCS cohort across Sweeps 1-6

(the cohort child ages 9 months, 3, 5, 7, 11 and 14) were selected (N=18,465). The

final analytic sample was created by keeping those who completed the Sweep 6 survey to apply an appropriate survey weight (N=11,252).'

In addition, the description of how the sample is distributed across UK countries is potentially misleading. How does this vary from the original sample distribution?

Measures

Page 8: The Kessler questions. Be clearer that the original 1-5 values are reverse coded to values 0-4, giving a score range of 0-24.

Predictor: Care experience.

This sentence needs to be revised as it is poorly phrased. Maybe include original question wording?

Mediators

Page 9: when detailing the measures include how they are scored (e.g. malaise 0'no' 1'yes' and include score range for each measure here.

When looking at MPD14 why only include prior mental health at child age 9 months not later on = when these are the same measure?

Social support – is question 1 reverse coded?

Page 10: which sweep was grandfather's/ grandmother's work status included. 9 months? State why included – as a measure of family SES?

Statistical analysis

I am not familiar with the statistical techniques used, so I suggest another reviewer should comment on the appropriateness of their application.

Page 10: I do not see the relevance of the inclusion of 'relative to the child population in the UK (e.g., 83,630 in England in 2024) [11]' I suggest amending to something like 'Whilst the overall sample size of this study was large, the care-experienced sample was small and the variable had imbalanced group cell sizes (See Table 1).

Page 11: I suggest placing the section on missingness and imputation earlier on –either in the data and methods section or before Statistical analysis. This is my main concern about the paper. You could include a supplementary table showing original v imputed descriptives, which should show that you have made the data more representative of the original sample, but in my view this is not essential. Or did you only impute to have a consistent sample size? You state 'missing data were assumed to be missing at random", but to maximise the plausibility of the MAR assumption the most important predictors of missing data need to be included in imputation model to further reduce bias and retain power. What auxiliary measures were included in your imputation model or was it just the analytic measures?

As the average missingness was 10%, imputing 10 datasets is correct. However, mention this specifically and cite Little & Rubin (2014) which explains this – include a sentence something like '…

based upon Rubin's Rule for the efficiency of estimation under a reported degree of missingness across the whole data of around 0.10 (Little and Rubin, 2014)'.

Little, R. & Rubin, D. (2014). Statistical Analysis with Missing Data. 2nd Edition, John Wiley & Sons, Hoboken.

Results

Page 12 - Table 1 and Table 2: as you use the imputed data in your analyses, this table should be based on imputed data and just show the means/proportions by care experience and either Ses or 95% CIs. I do not think significance testing is necessary in a descriptive table.

Page 16 – Table 3: it is not necessary to report SEs and CIs

L29 As shown in Figure 2 – this should be Figure 1?

Page 18: the section describing Table 4 is too detailed. All of the statistical results in the text are included in the table. Also, I think the language here needs to be clearer. Maternal OHC experience did not directly predict maternal distress at child age 14, once the mediators were included in the modelling. OHC predicted [e.g.] low income, which in turn predicted maternal distress at 14.

Page 19-21: Maybe Table 4 as supplementary and just use Figure 2? Figure 2 is great

In Table 4 and Figure 2 I am confused about the direction of the coefficient between prior mental health and MPD14? Are not high scores in both measures indicative of poor mental health? Thus shouldn't the coefficient be +ve? It is -0.68

Discussion

Page 21: 'their motherhood journey tended to start with higher rates of mental health

Difficulties'. From the analysis, we do not know if this the start of their motherhood journey as you have not included a measure of other children the mother already had when she had the MCS child. Maybe mention it as a limitation or an inclusion in further research? Also whether the mother is a single parent which adds to the burden of parenting….

**Reviewer**	**2**
**Name**	**Shin, Yongyun**
**Affiliation**	**Virginia Commonwealth University**
**Date**	**05-Jan-2026**
**COI**	**None**

This paper examines long-term growth trajectories of psychological distress among care-experienced and non-care-experienced mothers raising children from 9 months to 14 years of age. The children come from a nationally representative cohort of Great Britain, born between 2000 and 2002. The authors report that distress levels are consistently higher among care-experienced mothers and rise more steeply once children reach age 7, after adjusting for baseline covariates. They also show that the effect of care-experience is mediated by mother's baseline mental health, locus of control, household income, and social support measured at 9 months.

Overall, the findings appear important. However, some predictors may not be measured with high reliability, the results depend heavily on multiple imputation of missing values with associated concerns, and the clarity of presentation could be improved.

The Cronbach's α values for social support and locus of control are comparatively low at 0.52 and 0.62, respectively. Thus, these constructs consist of items that are weakly correlated and thus may not be measured reliably. This should at least be acknowledged as a limitation. Furthermore, these constructs, along with grand father's and mother's work statuses, have up to 41% missing values among 156 care-experienced mothers. The mediated effects at age 14, in particular, depend heavily on these imputed values. Each of these mothers' missing characteristics is imputed twice, once for the random coefficients model and once for the mediational model at child's age 14. That means two different values may be drawn for the same missing characteristic based on different models. However, because these models are related, and age 14 is part of the random coefficients model, multiple imputations based on this single model, while accommodating indirect effects, seem more appropriate. This limitation should be revised or at least explicitly noted in Discussion.

In Table 3, models 1-3 include different sets of interaction terms, yet the estimated variances and co-variances of the mother-specific random effects and the occasion-specific random error term remain exactly identical across models. This is unusual. Could the authors provide an explanation? Additionally, the footnote explains "Time1 indicates a rate of change in maternal distress from the child's age from 3 to 7 years. Time2 indicates a rate of change in maternal distress from the child's age of 7 to 14 years." This is potentially confusing because Time1 and Time2 appear to indicate the time period before and after age 7. If so, which specification is correct: 9 month $<$ Time1 $\leq$ age 7 and age 7 $<$ Time2 $\leq$ age 14, or 9 month $<$ Time1 $<$ age 7 and age 7 $\leq$ Time2 $\leq$ age 14?

A related interpretation concerns the increase in mothers' distress levels at Time2, which coincides with

early primary to upper primary shit. Could this increase be attributed to the schooling transition, in addition to Time2 coinciding with the Great Recession in the UK, as noted in the paper?

Other minor points are as follows:

1. line 7, page 6 of 55, ... than non-care-experienced ...

2. line -3, page 19 of 55, drop "fully through the maternal mediators", which is confusing when describing a direct effect

---

VERSION 1 - AUTHOR RESPONSE

Response to Reviewer 1

Abstract

1. I would suggest amending the 'outcomes' section to jjst include the outcomes – not the mediators.

Response: Thank you for the comment. As suggested by the reviewer, we have only included the outcomes in the Outcome section and amended the text in the following manner: "*The primary outcomes were psychological distress of mothers at child ages 3, 5, 7, 11 and 14.* on page 2.

2. 'into their child's development'. Should this be 'over their child's development'?

Response: After consideration of your comment, we have changed "*care-experienced mothers were more likely to experience distress that persisted into their child's development"* to "*care-experienced mothers were consistently more likely to experience distress across child ages 3 to 14*". We believe this change better reflects our results, which show disparities in maternal distress at age 3 and that this remains stable across child ages 3 to 14.

Strengths and limitations

3. Representativeness cannot be assumed for mothers. This is correct, but maybe add that this 'representativeness' will almost certainly particularly apply to the care-experienced sample of mothers. Many of the mothers initially contacted would potentially be more likely to not take part if they have out-of-home care experience.

Response: Thank you for the insightful comment. As the reviewer suggested, we have added the following text to clarify the underrepresentation of the care-experienced population in surveys on page 3: "*and especially the care-experienced group is generally underrepresented in population surveys*."

Background

4. 'care experience' 'social care' 'out-of-home-care' are all used. It would help the reader if one is used. I suggest out-of-home care experience or OHC

Response: As per the reviewer's comment, we have ensured that the term, *care experience*, is consistently used throughout the main manuscript. We have also highlighted the use of this term by mentioning that "*hereafter, out-of-home care is referred to as care experience*" on page 4.

We have used this term to refer to out-of-home care, as our care-experienced lived experience advisor preferred it, as it is perceived as less stigmatising and more inclusive and person-centred. Additionally, it better reflects their experience in the care system. Hence, we have provided our rationale for using this term in the footnote.

5. Page 4: Ref 12. Please check that child abuse and neglect are still the main reasons children go into care in the UK? This reference is quite old (2013) and I think there are increasingly older and unaccompanied (migrant) children in the care system.

Response: We thank the reviewer for raising this point. Yes, according to the NSPCC and England government statistics, abuse and neglect are still the most common reasons for entry to care, although we do recognise that the population of children in the care system has changed over time, and now includes more adolescents and unaccompanied children. We agree that the reference (2013) is outdated, so we have added new references for the latest NSPCC report published in 2024 and the England government's children looked after statistics published in 2025 on page 4 and in the reference section.

6. Into childhood and adolescence. Amend to 'through childhood and into adolescence'?

Response: We have revised the text to "*through childhood and into adolescence*", as the reviewer suggested on page 4.

7. Include 'measure' after distress and include what the measure is and reference it?
   Malaise Inventory
   Rutter, M., Tizard. J., & Whitmore, K. (1970). Education, health and behaviour. London: Longman.
   Rodgers, B., Pickles, A., Power, C., Collishaw, S. & Maughan, B. (1999). Validity of the Malaise Inventory in general population samples. Social Psychiatry and Psychiatric Epidemiology, 34(6), 333-341. https://doi.org/10.1007/s001270050153

Response: Thank you. We have added the aforementioned references in the reference section and in the main text in the following manner on page 4: “*Emerging evidence suggests that care-experienced mothers tend to face increased risks of experiencing psychological distress [41,83] than non-care-experienced mothers within the first year after birth [9,10].*”

8. Page 5: ‘as parenthood places’ change to ‘as parenthood demands’?

Response: We have amended the text to “*as parenthood demands different physical and mental investments and rewards for parents*”, as the reviewer suggested on page 5.

9. Page 6: find a better word for ‘inquiries’

Response: We have replaced “*inquiries*” with “*knowledge gaps*” on page 6, as the reviewer suggested.

10. I am unconvinced about the inclusion of a child’s birthweight as a mediator of psychological distress.

Response: We thank the reviewer for raising this question. We included a child’s birthweight as a mediator, as the study by Botchway and associates (2014) used the same MCS data and shows that care-experienced mothers tended to have lower birthweight babies. A child’s low birthweight, in turn, was found to be associated with increased maternal psychological distress in the general population (Greene et al., 2019; Singer et al., 1999). We appreciate that this would not necessarily be the strongest and most consistent factor that explains the increased psychological distress of care-experienced mothers across their child’s childhood and adolescence. Nevertheless, given the aforementioned literature and our study’s aim of exploring potential postpartum-associated pathways, we think that testing the effect of this birthweight factor as an intermediate perinatal factor is justifiable and beneficial, to show whether this can partly explain the greater psychological distress of care-experienced mothers in the early years, relative to those without care experience.

See the references:

Botchway SK, Quigley MA, Gray R. Pregnancy-associated outcomes in women who spent some of their childhood looked after by local authorities: findings from the UK Millennium Cohort Study. BMJ Open. 2014;4(12):e005468.

Greene MM, Schoeny M, Rossman B, Patra K, Meier PP, Patel AL. Infant, maternal, and neighborhood predictors of maternal psychological distress at birth and over very low birth weight infants' first year of life. J Dev Behav Pediatr. 2019;40(8):613–21. doi: 10.1097/DBP.0000000000000704.

Singer LT, Salvator A, Guo S, Collin M, Lilien L, Baley J. Maternal psychological distress and parenting stress after the birth of a very low-birth-weight infant. jama. 1999 Mar 3;281(9):799-805.

Methods

11. Page 7: the n=19243 is incorrect for the age 9 month sample. This is n=18,818 children and n=18,552 families.

Response: Thank you for the comment and identification of this error. We have corrected this on page 7.

12. I am unclear about the following sentence and resulting sample size which is then restricted to those who participated at age 14. This needs to be made much clearer to me.
    'Out of the original sample at Sweep 1 (N=19,243), birth mothers who provided consistent information about their relationship to the MCS cohort across Sweeps 1-6
    (the cohort child ages 9 months, 3, 5, 7, 11 and 14) were selected (N=18,465). The
    final analytic sample was created by keeping those who completed the Sweep 6 survey to apply an appropriate survey weight (N=11,252).'

Response: We thank the reviewer for raising this point. We have clarified and revised the text about the selection of the analytic sample on page 7 in the following manner, as the reviewer suggested:

"The analytic sample for this study was created in two steps. First, out of the original sample at Sweep 1 (N=18,552), we selected birth mothers who provided consistent information about their relationship to the MCS cohort across Sweeps 1-6 (the cohort child ages 9 months, 3, 5, 7, 11 and 14) (N=18,465). We then further restricted the sample to those who completed the Sweep 6 survey to apply a survey weight (N=11,252)."

13. In addition, the description of how the sample is distributed across UK countries is potentially misleading. How does this vary from the original sample distribution?

Response: As the reviewer requested, we have checked that the distribution of the original sample (N=18552) across the UK countries was similar to that of the final analytic sample (N=11252), as reported on page 7. We have also cross-validated this against the distribution reported in the Millennium Cohort Study User Guide (Surveys 1-5).

Reference

Centre for Longitudinal Studies (CLS). Millennium Cohort Study User Guide (Surveys 1–5). London: Centre for Longitudinal Studies; 2020.

Measures

14. Page 8: The Kessler questions. Be clearer that the original 1-5 values are reverse coded to values 0-4, giving a score range of 0-24.

Response: Thank you for the comment. As suggested by the reviewer, we have revised the text in the following manner on page 8:

"the original values were reverse-coded on a scale from 0 to 4, producing a total range of 0 to 24 and higher values indicate higher distress."

In addition, we have clarified the text by changing "*6-item Likert scale*" to "*6-point Likert scale*". We have also kept the original wording of 'can't say' from the survey questionnaire, instead of mentioning "cannot say".

15. Predictor: Care experience.
    This sentence needs to be revised as it is poorly phrased. Maybe include original question wording?

Response: We have revised "*Predictor: Care experience*" to "*Maternal care experience in childhood*" to be clear about the predictor, as the reviewer suggested on page 8. As the reviewer suggested, we have included the original wording of the questionnaire.

Mediators

16. Page 9: when detailing the measures include how they are scored (e.g. malaise 0'no' 1'yes' and include score range for each measure here.

Response: Thank you for the comment. We have explained how each measure is scored and its range in the following manner on pages 9 and 10, as suggested by the reviewer:

On pages 9 and 10,

"Mediators included the continuous measure of the self-reported MCS child's birth weight (kilos) as well as the following maternal factors collected when the MCS child was 9 months old."

"This scale includes nine binary items (1=yes, 0=no) about how often an individual experiences the following symptoms:" … "). A total score was used by summing scores from the nine items. Higher scores indicate higher mental health difficulties, with a range of 0 to 9."

"Maternal perceived social support: Perceived social support was originally measured using the three items on a 6-point scale (strongly agree (1) to strongly disagree (5) and can't say (6)), including (a) I have no one to share feelings with, (b) I have other parents to talk to, and (c) family would help if I had financial problems. 'Can't say' response was treated as missing. (b) and (c) statements were reverse-coded so that higher values indicate higher perceived support. The summed scores were used for analysis (Cronbach's α = 0.52), resulting in a total range of 3 to 1.."

"Maternal locus of control: Locus of control was largely defined as one's perceived control over one's life and was originally measured on a 3-point scale by asking (a) whether respondents get what they want out of life (b) whether they have control over life and (c) whether they run life the way they want (See the supplementary document for the full response categories). 'Can't say' was treated as missing. Reverse coding was applied to (b) and (c) statements, and summed scores (range of 3-6) were used to indicate that higher values mean feeling more in control (Cronbach's α = 0.62)."

17. When looking at MPD14 why only include prior mental health at child age 9 months not later on = when these are the same measure?

Response: Thank you for the comment. We examined whether prior mental health at the child's age 9 mediates the association between maternal care experience and their distress at the child's age 14. This is because we align with our aim to investigate postpartum factors (measured within the first year of the MCS child) that may explain disparities in maternal distress at child age 14 based on care experience.

18. Social support – is question 1 reverse coded?

Response: We reverse-coded the second and third statements so higher values indicate higher perceived support.

19. Page 10: which sweep was grandfather's/ grandmother's work status included. 9 months? State why included – as a measure of family SES?

Response: We used the earliest available grandfathers' and grandmothers' information from the Sweep 2 survey as a proxy measure of maternal grandparents' socioeconomic status. We have revised the text to be clear about this on page 10: "*The earliest available information from Sweep 2 on maternal grandmother's and maternal grandfather's work status when the mother was 14 years old was used as a proxy measure of maternal grandparents' socioeconomic status*."

Statistical analysis

20. Page 10: I do not see the relevance of the inclusion of 'relative to the child population in the UK (e.g., 83,630 in England in 2024) [11]' I suggest amending to something like 'Whilst the overall sample size of this study was large, the care-experienced sample was small and the variable had imbalanced group cell sizes (See Table 1).

Response: We have revised the text as the reviewer suggested on page 11: "*Whilst the overall sample size of this study was large, the care-experienced population size is small, and the care experience variable had imbalanced group cell sizes (See Table 1)*."

21. Page 11: I suggest placing the section on missingness and imputation earlier on –either in the data and methods section or before Statistical analysis.

Response: Thank you for the thoughtful suggestion. As recommended by the reviewer, we have discussed the missingness and imputation before presenting statistical analyses on page 10. We signposted readers to a later section for details on our multiple imputation approaches, as these are closely tied to the specification of the analytic models, which need to be introduced first.

22. You could include a supplementary table showing original v imputed descriptives, which should show that you have made the data more representative of the original sample, but in my view this is not essential.

Response: The requested information is available in the Supplementary Table 1 in the supplementary document.

23. Or did you only impute to have a consistent sample size? You state 'missing data were assumed to be missing at random", but to maximise the plausibility of the MAR assumption the most important predictors of missing data need to be included in imputation model to further reduce bias and retain power. What auxiliary measures were included in your imputation model or was it just the analytic measures?

Response: We thank the reviewer for the comment. We mainly imputed the data to reduce bias and retain power for statistical analyses as much as possible. We only included the analytic variables in the multiple imputation models, as we explained in the manuscript, the average missingness across all variables included in the models is low - approximately 10%. The analytic variables included in the imputation models strongly predicted the probability of missingness in most of the variables with missing values, which well satisfied the Missing at Random (MAR) assumption. Especially, the analytic variables were strongly associated with the missingness of maternal distress outcome variables, as well as that of mediators, including locus of control and social support, which relatively showed a higher percentage of missingness than other mediators, although their missingness is moderate in population social surveys.

24. As the average missingness was 10%, imputing 10 datasets is correct. However, mention this specifically and cite Little & Rubin (2014) which explains this – include a sentence something like '... based upon Rubin's Rule for the efficiency of estimation under a reported degree of missingness across the whole data of around 0.10 (Little and Rubin, 2014)'.
Little, R. & Rubin, D. (2014). Statistical Analysis with Missing Data. 2nd Edition, John Wiley & Sons, Hoboken.

Response: Thank you for the comment and reference. We have revised the text as the reviewer suggested on page 11 in the following manner:

“10 imputed datasets were generated based on Rubin’s Rules [85] to estimate the uncertainty introduced by missing data, given the missingness in our data.”

Results

25. Page 12 - Table 1 and Table 2: as you use the imputed data in your analyses, this table should be based on imputed data and just show the means/proportions by care experience and either Ses or 95% CIs. I do not think significance testing is necessary in a descriptive table.

Response: We thank the reviewer for this helpful suggestion. As requested by the reviewer, we have presented the means/proportions by care experience and their SE in Supplementary Table 1 in the supplementary document. Our inferential analyses are based on imputed datasets derived from two separate multiple imputation models, reflecting different data structures required for the statistical models, as explained in the Statistical analysis section. Presenting descriptive statistics based on the imputed data in the main table would therefore require reporting estimates for each set of imputed datasets, which we felt could reduce clarity and lead to confusion for readers. This is exemplified by reporting two different pooled estimates from the imputed datasets for the same control variables.

Hence, to balance transparency and clarity, we present the descriptive profiles of the analytic sample in Tables 1 and 2 based on the observed data. At the same time, the corresponding estimates derived from the imputed datasets (including means and proportions by care experience), which are requested by the reviewer, are provided in Supplementary Table 1. We have signposted readers to Supplementary Table 1 for the descriptive statistics based on the imputed data in the main text on page 12. This approach allows us to clearly describe the sample characteristics while ensuring that results based on the imputed data are fully reported.

26. Page 16 – Table 3: it is not necessary to report SEs and CIs

Response: We have reported CIs only in Tables 2 and 3, as the reviewer suggested.

27. L29 As shown in Figure 2 – this should be Figure 1?

Response: Thank you for spotting this error. We corrected the numbering of the figures on page 16, as the reviewer suggested.

28. Page 18: the section describing Table 4 is too detailed. All of the statistical results in the text are included in the table. Also, I think the language here needs to be clearer. Maternal OHC experience did not directly predict maternal distress at child age 14, once the mediators were included in the modelling. OHC predicted [e.g.] low income, which in turn predicted maternal distress at 14.

Response: Thank you for the comment. We streamlined the text describing the results to enhance clarity on page 19, as the reviewer suggested:

"Partially confirming hypothesis 2, maternal care experience did not directly predict maternal distress at child age 14 once all the postpartum-associated mediators were included in the model ($B_{Care\ experience \rightarrow MPD14}$ = 1.08, 95% CI [-0.44,2.61], p = 0.163). As shown in Figure 2, Part of the effect of maternal care experience on maternal distress at the child's age 14 was mediated through the postpartum factors. Mothers with care experience were more likely to experience greater mental health difficulties ($B_{Care\ experiene \rightarrow Prior\ mental\ health \rightarrow MPD14}$ = 0.57, 95% CI [0.18,0.96], p = 0.005), lower locus of control ($B_{Care\ experience \rightarrow Locus\ of\ control \rightarrow MPD14}$ = 0.31, 95% CI [0.11,0.51], p = 0.002), lower household income ($B_{Care\ experience \rightarrow Household\ Income \rightarrow MPD14} = 0.28$, 95% CI [0.15,0.41], p < 0.001), lower social support ($B_{Care\ experience \rightarrow Social\ Support \rightarrow MPD14}$ = 0.16, 95% CI [0.05,0.28], p = 0.006) at 9 months postpartum, which, in turn, predicted higher maternal distress at child age 14."

29. Page 19-21: Maybe Table 4 as supplementary and just use Figure 2? Figure 2 is great
    In Table 4 and Figure 2 I am confused about the direction of the coefficient between prior mental health and MPD14? Are not high scores in both measures indicative of poor mental health? Thus shouldn't the coefficient be +ve? It is -0.68

Response: We appreciate the reviewer's helpful comment. We have retained both Figure 2 and Table 3 (Formerly Table 4, now renumbered after combining the previous Tables 1 and 2), as both provide key complementary information. Table 3 provides the full set of key results, including unstandardised estimates, 95% confidence intervals and proportion mediated. Whereas Figure 2 offers a visual representation of these effects, enabling readers to interpret the relations more easily between variables.

We also appreciate the reviewer's observation regarding the direction of the prior mental health effect. In this original analysis, a reverse-coded version of this measure was used so that all mediators had the consistent direction of the association with care experience. Nevertheless, we recognise that this was unnecessary, and we have therefore refitted the parallel mediation model with the original coding of the prior mental health measure (i.e., higher values, higher prior mental health difficulties). The results showed the positive relationship between care experience and prior mental health, as well as between prior mental health and maternal distress at the child's age 14, as we originally observed. We have revised Table 3, Figure 2, and the Results section in the abstract, accordingly, to reflect the updated results.

Discussion

30. Page 21: 'their motherhood journey tended to start with higher rates of mental health Difficulties'. From the analysis, we do not know if this the start of their motherhood journey as you have not included a measure of other children the

mother already had when she had the MCS child. Maybe mention it as a limitation or an inclusion in further research? Also whether the mother is a single parent which adds to the burden of parenting....

Response: We thank the reviewer for this insightful comment. We agree with the reviewer's point and revised the text to "*their motherhood journey tended to be characterised by higher rates of mental health difficulties*" on page 22 and "*This finding underscores the importance of continuous mental health support, especially during motherhood*" on page 22.

We have also encouraged readers to consider single parenthood and the number of children to examine variations in maternal distress among care-experienced mothers in the Strengths, limitations, and future directions section.

Here is the revised text:

"antecedents and consequences of heightened maternal distress among care-experienced mothers can be examined by considering maternal characteristics (e.g., early educational and health histories, single parenthood, the number of children, and the intersection of ethnicity and care experience [69]),"

Response to Reviewer 2

1. The Cronbach's α values for social support and locus of control are comparatively low at 0.52 and 0.62, respectively. Thus, these constructs consist of items that are weakly correlated and thus may not be measured reliably. This should at least be acknowledged as a limitation. Furthermore, these constructs, along with grand father's and mother's work statuses, have up to 41% missing values among 156 care-experienced mothers.

Response: We thank the reviewer for the important comments. As suggested by the reviewer, we fully acknowledged the limitations of those measures in the Discussion section on pages 25 and 26 in the following manner:

"our social support and locus of control measures showed low reliability, and therefore, it is important to replicate the analysis with alternate measures."

"Meanwhile, future research is advised to use better quality measures to control for the care-experienced mothers' socioeconomic status in childhood, given the relatively high missingness in their grandparents' work status measures."

2. The mediated effects at age 14, in particular, depend heavily on these imputed values. Each of these mothers' missing characteristics is imputed twice, once for the random coefficients model and once for the mediational model at child's age 14. That means two different values may be drawn for the same missing

characteristic based on different models. However, because these models are related, and age 14 is part of the random coefficients model, multiple imputations based on this single model, while accommodating indirect effects, seem more appropriate (I do not understand this comment). This limitation should be revised or at least explicitly noted in Discussion.

Response: We thank the reviewer for highlighting consistency across imputations. As requested by the reviewer, we acknowledged the aforementioned limitation in the Discussion section on page 25 in the following manner:

"As explained in the Statistical analysis section, we used the two separate multiple imputation models, each specified to be congenial with its corresponding statistical model, to obtain valid statistical inference. Nevertheless, this approach generated different imputed values for the same missing observation, although the overall distributions of these values were similar (See Supplementary Table 1). Additionally, the estimates from each statistical model were not directly comparable, as different sets of imputed datasets were used."

We conducted two different multiple imputation models corresponding to each analytic model - multilevel multiple imputation for the random slope (coefficients) model and single-level multiple imputation for the parallel mediation model. This was to ensure each multiple imputation model preserves all the structural relationships that each analytic model estimates. As explained in the Statistical analysis section on page 11, these two analytic models were based on different structures of the data and outcomes, although the same MCS data was used. Random slope model used multilevel data where repeated distress outcomes from child age 3 to 14 were clustered within an individual. Whereas the parallel mediation model used single-level data, predicting a single distress outcome at child age 14. Ignoring the clustering in the multilevel data and imputing the data using a single-level multiple imputation are known to underestimate the intraclass correlation, introducing bias in estimating regression coefficients and intraclass correlation (Van Burren, 2018; Enders et al., 2016; Grund et al., 2016). This is particularly relevant to our study, given the relatively high intraclass correlation in the distress outcomes and our interest in estimating variance components. At the same time, the multilevel multiple imputation incorporates random effects that are not part of the single-level parallel mediation model, and this imputation model would be more complex than necessary for this analytic model. For these reasons, we used two separate multiple imputation models, so each imputation model is congenial with the analytic model. A similar multiple imputation approach is also used in other research (e.g., Huss et al., 2022).

We truly understand the reviewer's point on having two different values for the same missing characteristic. Our philosophy of multiple imputation is not to recover the true missing value for the missing characteristic, but to generate plausible values drawn from the predictive distribution of missing data to produce valid parameter estimates. In this approach, different imputation models may generate different plausible values for the same missing observation. We believe that this would not compromise the validity of statistical estimates as long as the imputation model is compatible with the corresponding analytic model. Nevertheless, we appreciate that the estimates from

each analytic model are not based on the same imputed datasets, so they are not directly comparable. Thus, we have acknowledged this limitation in the Discussion section to be transparent about our imputation approach, as suggested by the reviewer. We have also clarified our multiple imputation approach on page 11.

References

van Buuren, S. (2018). Flexible Imputation of Missing Data, Second Edition (2nd ed.). Chapman and Hall/CRC. https://doi.org/10.1201/9780429492259

Enders CK, Mistler SA, Keller BT. Multilevel multiple imputation: A review and evaluation of joint modeling and chained equations imputation. Psychol Methods 2016 06;21(2):222-240.

Grund S, Lüdtke O, Robitzsch A. Multiple imputation of missing data for multilevel models: Simulations and recommendations. Organizational Research Methods. 2018 Jan;21(1):111-49.

Huss L, Butt ST, Borgquist S, Elebro K, Sandsveden M, Manjer J, Rosendahl A. Levels of vitamin D and expression of the vitamin D receptor in relation to breast cancer risk and survival. Nutrients. 2022 Aug 16;14(16):3353.

3. In Table 3, models 1-3 include different sets of interaction terms, yet the estimated variances and co- variances of the mother-specific random effects and the occasion-specific random error term remain exactly identical across models. This is unusual. Could the authors provide an explanation?

Response: Thank you for the comment. The estimated variances and covariances were not identical across Models 1-3. These differences are not visible in the table because the values were rounded to two decimal places. I updated the note for Table 3 to be clear about this in the following manner:

"Variances and covariances were not identical across Models, and they appeared so due to rounding the values to two decimal places."

4. Additionally, the footnote explains "Time1 indicates a rate of change in maternal distress from the child's age from 3 to 7 years. Time2 indicates a rate of change in maternal distress from the child's age of 7 to 14 years." This is potentially confusing because Time1 and Time2 appear to indicate the time period before and after age 7. If so, which specification is correct: 9 month < Time1 ≤ age 7 and age 7 < Time2 ≤ age 14, or 9 month < Time1 < age 7 and age 7 ≤ Time2 ≤ age 14?

Response: As explained in the Statistical analysis section, we fitted two linear splines with a knot point of age 7. This allowed us to estimate two slopes, with the first one from

age 3 to 7 and the second one from age 7 to 14. Age 7 is the reference point where the two linear spline segments join. Thus, the interval notation is the following:

$$\text{Time1: } 3 \leq time \leq 7$$

$$\text{Time2: } 7 \leq time \leq 14$$

5. A related interpretation concerns the increase in mothers' distress levels at Time2, which coincides with early primary to upper primary shift. Could this increase be attributed to the schooling transition, in addition to Time2 coinciding with the Great Recession in the UK, as noted in the paper?

Response: Thank you for the reviewer's thoughtful suggestion. Yes, we agree that this could be a potential reason for the increased vulnerability to distress after age 7. As suggested by the reviewer, we have revised the text to make this point clearer in the following manner on page 22:

"Increased vulnerability to distress was shown among both mother groups as their child entered adolescence. This might be influenced by shifts in developmental and environmental demands (e.g., school transition) and parent-child relationship dynamics [25,26], although a careful interpretation was needed as this coincided with the Great Recession in the UK [47]."

Other minor points are as follows:

6. 1. line 7, page 6 of 55, ... than non-care-experienced ...

Response: Thank you for the comment. We have revised the text to "*non-care-experienced mothers*", as suggested by the reviewer.

7. 2. line -3, page 19 of 55, drop "fully through the maternal mediators", which is confusing when describing a direct effect

Response: Thank you for the comment. We have revised the text to be clearer about the direct effect, as the reviewer suggested, in the following manner:

"maternal care experience did not directly predict maternal distress at child age 14 once all the postpartum-associated mediators were included in the model."

Unrequested changes

1. We have corrected how we estimated standard errors for the estimates from the parallel mediation model on page 11 in the following manner:

Table 1 Comparison between original and revised text

Page	Original text	Revised text
11	The bootstrapping method was used to produce less biased estimates of standard errors with unequal group size [46]	maximum likelihood estimation with robust (Huber-White) standard errors and a scaled test statistic that is asymptotically equal to the Yuan-Bentler test statistic [46] was used.<br><br>We have also updated the reference number 46

2. We have made the following minor revisions to enhance the clarity of the text without altering its substantive meaning and arguments. Please see the document - marked copy to find all revisions and edits. Table 2 shows the comparison between the original and revised text. The revisions are highlighted in red in the text.

Table 2 Comparison between original and revised text

Page	Original text	Revised text
2	This study aims to examine (a) disparities in trajectories of psychological distress in care-experienced versus non-care-experienced mothers whilst raising their children from childhood to adolescence, and (b) putative pathways shaping these disparities in maternal distress.	This study aims to explore (a) disparities in trajectories of psychological distress in care-experienced versus non-care-experienced mothers as they raise their child from age 3 to 14, and (b) putative postpartum-associated pathways shaping disparities in maternal distress at child age 14.
2	The primary outcomes were psychological distress of mothers at the child ages of 3, 5, 7, 11 and 14.	The primary outcomes were psychological distress of mothers at child ages 3, 5, 7, 11 and 14.
3	This study is the first attempt to provide life course insights into long-term disparities in maternal psychological distress based on care experience and putative pathways driving these disparities in the UK.	This study is the first attempt to provide life course insights into long-term disparities in maternal psychological distress based on out-of-home care experience and putative postpartum pathways shaping distress disparities
3	Our new evidence of the potential pathway from maternal care experience to maternal distress uniquely indicates areas for strengthening the maternal and	We deleted this point as this was not related to the method of this study.

	early childhood public health interventions.	
4	out-of-home care refers to children who is involved with child welfare services	out-of-home care refers to children who are involved with child welfare services
4	we examine long-term disparities in maternal psychological distress whilst raising their children into childhood and adolescence and potential postpartum-associated pathways driving these disparities.	we examine long-term disparities in maternal psychological distress between mothers with and without care experience as they raise their child through childhood and into adolescence. We also explore putative postpartum-related pathways that may shape maternal mental health disparities during their child's adolescence.
4	Particularly, emerging evidence suggests that care-experienced mothers tend to face increased risks of experiencing psychological distress	Emerging evidence suggests that care-experienced mothers tend to face increased risks of experiencing psychological distress
5	In this context, care-experienced mothers might experience more distress than care-experienced mothers over time. Qualitative findings highlight	Care-experienced mothers, especially younger women, might experience more vulnerability to distress than non-care-experienced mothers. Qualitative findings suggest that
5	to date, it is unknown how disparities in distress trajectories unfold over time between mothers with and without care experience throughout childhood and adolescence.	to date, it is unknown how disparities in distress trajectories unfold over time between mothers with and without care experience throughout their child's childhood and adolescence.
5	Additionally, it remains unclear about the potential pathways through which care experience shapes maternal psychological distress.	Additionally, it remains unclear about the potential postpartum-associated pathways through which maternal care experience shapes maternal psychological distress during their child's adolescence.
5	Whilst childhood adversities do not inevitably translate into adult psychopathology [27], as the life course perspective suggests, early adversities can lead to a chain of social disadvantages across life domains over time. This can, in turn, exacerbate mental health difficulties later in life [30,31].	Childhood adversities do not inevitably translate into adult psychopathology [27]. However, the life course perspective suggests that early adversities can lead to a chain of social disadvantages across life domains over time, which can, in turn, exacerbate mental health difficulties later in life [30,31].
5	The care experience in childhood is	The maternal care experience in childhood is

6	[32,33,34,35]	[32,33,34,35,84]
6	it is uncertain what roles these factors play in shaping the distress of care-experienced mothers in the long term.	it is uncertain what roles these factors play in shaping the distress of care-experienced mothers during their child's adolescence.
6	the UK's longitudinal Millennium Cohort Study (MCS) data collected at the child's ages of 9 months, 3, 5, 7, and 14 years [38].	the UK's longitudinal Millennium Cohort Study (MCS) data collected at child ages 9 months, 3, 5, 7, 11 and 14
6	how maternal distress trajectories vary between mothers with and without care experience, from the child's ages 3 to 14	how maternal distress trajectories vary between mothers with and without care experience, across child ages 3 to 14
6	the relationship between mothers' childhood care experience and heightened maternal distress at the child's age of 14	the relationship between mothers' childhood care experience and heightened maternal distress at child age 14
8	Items concern how often individuals feel hopeless, restless/fidgety, that everything is an effort, worthless, and nervous.	Items concern how often individuals feel depressed, hopeless, restless/fidgety, that everything is an effort, worthless, and nervous.
10	To examine the trajectories of maternal distress between mothers with and without care experience from the child's age of 3 to 14 years	To examine the trajectories of maternal distress between mothers with and without care experience across child ages 3 to 14 years
10	Hence, we fitted two splines with a knot point of age 7, to separately capture a pattern of change before and after age 7.	Hence, we fitted two linear splines with a knot point of age 7, to separately capture a pattern of change before and after age 7.
11	to examine the effects of multiple mediators in the relationship between maternal care experience and maternal distress at the child's age 14,.	to examine the effects of multiple mediators in the relationship between maternal care experience and maternal distress at child age 14,
11	A single-level multiple imputation with 10 imputed datasets was conducted for the parallel mediation analysis	A single-level multiple imputation with 10 imputed datasets was conducted for the parallel mediation model
12	Bivariate correlation results indicated that repeated maternal distress scores from the MCS child's ages 3 to 14	Bivariate correlation results indicated that repeated maternal distress scores
12	In line with the baseline characteristics, maternal care experience in childhood was associated with the outcomes and mediators in the expected manner	Maternal care experience in childhood was associated with the outcomes and mediators in the expected manner

12	Additionally, maternal care experience was correlated with elevated distress at each child's ages of 3, 5, 7, 11 and 14 (Table 2, Supplementary Table 3).	Additionally, maternal care experience was correlated with elevated distress at child ages 3, 5, 7, 11 and 14 (Table 1, Supplementary Table 3).
12	These mediators were associated with higher distress scores at the child's age of 14	These mediators were associated with higher distress scores at child age 14
12	Table 1 Baseline sample characteristics (at the child's of age 9 months)	Table 1 Baseline sample characteristics (at child age 9 months)
16	Table 2 presents results from a series of piecewise mixed effects models.	Table 2 presents results from a series of piecewise random slope models.
17	). The final results from Model 3 suggest that maternal distress, on average, did not significantly change before the child's age of 7 …<br>mothers' distress levels, on average, tended to increase after the child's age of 7	). The final results from Model 3 suggest that maternal distress, on average, did not significantly change before child age 7<br>…<br>mothers' distress levels, on average, tended to increase after child age 7
18	**Note:** Time1 indicates a rate of change in maternal distress from the child's ages of 3 to 7. Time2 indicates a rate of change in maternal distress from the child's ages of 7 to 14.	**Note:** Time1 indicates a rate of change in maternal distress from child age 3 to 7. Time2 indicates a rate of change in maternal distress from child age 7 to 14.
21	MPD14 = Maternal psychological distress at the child's age of 14<br>…<br>Pooled results for 10 imputed datasets are reported.<br>…<br>The controls, including maternal age, ethnicity, maternal grandmother's and maternal grandfather's work status, were adjusted for.	MPD14 = Maternal psychological distress at child age 14<br>…<br>Pooled results for 10 imputed datasets based on Rubin's rules are reported.<br>…<br>The controls, including maternal age, maternal ethnicity, maternal grandmother's and maternal grandfather's work status, were adjusted for.
21	we explored disparities in the trajectories of psychological distress between mothers with and without care experience from the child's age of 3 to 14 years, and potential postpartum-associated pathways shaping these disparities.	we explored disparities in the trajectories of psychological distress between mothers with and without care experience across child ages 3 to 14, and potential postpartum-associated pathways shaping disparities at child age 14.
24	the mediating effect of social support in the relationship between care experience and maternal distress at the child's age of 14 was significant	the mediating effect of social support in the relationship between care experience and maternal distress at child age 14 was significant

	... Nevertheless, our evidence points to lower social support within one year of birth as one of the pathways to higher distress in care-experienced mothers at the child's age of 14.	... Nevertheless, our evidence points to lower social support within one year of birth as one of the pathways to higher distress in care-experienced mothers at child age 14.
25	antecedents or variations in maternal distress among care-experienced mothers can be better examined considering broad maternal characteristics, such as maternal early educational and health histories, ethnicity [69], whilst further examining their consequences on other health outcomes (e.g., premature mortality) [70, 80] or their child's outcomes.	antecedents and consequences of heightened maternal distress among care-experienced mothers can be examined by considering maternal characteristics (e.g., early educational and health histories, single parenthood, the number of children, and the intersection of ethnicity and care experience [69]), other health outcomes (e.g., premature mortality) [70, 80], and child's outcomes
26	Our mediation evidence suggests that attention should be paid to	Our mediation evidence suggests that attention can be paid to
12	All analyses were conducted in R programming, using mice, lme4, and lavaan packages.	All analyses were conducted in R programming, using mice, lme4, lavaan, and lavaan.mi packages.
18	Pooled estimates are reported for 10 imputed datasets.	Pooled estimates and uncertainty are reported for 10 imputed datasets.

VERSION 2 - REVIEW

**Reviewer**	**1**
**Name**	**Parsons, Sam**
**Affiliation**	**UCL IOE, Centre for Longitudinal Studies**
**Date**	**20-Apr-2026**
**COI**	

I would like to thank the authors for thoroughly addressing each of the points raised in my review of their manuscript. I only have a few further points that require minor clarification, after which I recommend publication. In response to reviewers document:

12. Additional reviewer comment:

I still find the sample derivation a little confusing in particular "we selected birth mothers who provided consistent information about their relationship to the MCS cohort across

Sweeps 1-6 (the cohort child ages 9 months, 3, 5, 7, 11 and 14) (N=18,465)" Do you mean consistent information was provided by birth mothers in the sweeps that they participated in? If this is so then please clarify this point. At the moment it suggests (to me) that the sample provided consistent information across S1 – S6, which is not the case given the n=18,465 sample size.

13. The response to the reviewer is great. Please add in to the text that the distribution of sample across UK is very representative - and add reference provided. The reader needs to know it is representative.

15. The original wording is not included in the paper as far as I can see? if original wording is not being used, what is there needs further clarification. Need to include that the CM mother did not live with her parents at some point before age 17.

16. I have amended text to be the EXACT wording; Prior maternal mental health: Scores from the modified Rutter Malaise Scale [41,42] were used to indicate the prior maternal mental health at 9 months postpartum. This scale includes nine binary items (1=yes, 0=no) about an individual's experience of the following symptoms: (1) 'feel tired most of the time', (2) 'often feel miserable or depressed', (3) 'often get worried about things', (4) 'often get into a violent rage', (5) 'often suddenly become scared for no good reason', (6) 'easily upset or irritated', (7) 'constantly keyed up or jittery', (8) 'every little thing gets on nerves and wears you out', (9) 'heart often races like mad' ....

24. Suggest including the level of missingness in your dataset for clarity for the reader. Include text in capitals - or something similar. "10 imputed datasets were generated based on Rubin's Rules [85] to estimate the uncertainty introduced by missing data, given the OVERALL LEVEL OF 10% missingness in our data."

**Reviewer**	**2**
**Name**	**Shin, Yongyun**
**Affiliation**	**Virginia Commonwealth University**
**Date**	**04-May-2026**
**COI**	

The revision improves clarity over the original submission, and the authors addressed my comments well.

VERSION 2 - AUTHOR RESPONSE

Response to Reviewer 1

12. I still find the sample derivation a little confusing in particular "we selected birth mothers who provided consistent information about their relationship to the MCS cohort across Sweeps 1-6 (the cohort child ages 9 months, 3, 5, 7, 11 and 14) (N=18,465)" Do you mean consistent information was provided by birth mothers in the sweeps that they participated in? If this is so then please clarify this point. At the moment it suggests (to me) that the sample provided consistent information across S1 – S6, which is not the case given the n=18,465 sample size.

Response: Thank you for such a helpful comment. Yes, that is exactly what we meant. Some mothers had inconsistent information about their relationship to the MCS child reported across sweeps. For instance, their relationship status was recorded as unknown at the subsequent sweep due to not being interviewed, despite being reported as a natural mother at Sweep 1. Hence, we kept mothers who reported their relationship status consistently in the sweeps they took part.

As the reviewer suggested, we have updated the text in the following manner:

"*we selected birth mothers who provided consistent information about their relationship to the MCS cohort in the sweeps in which they participated (N=18,465)*"

13. The response to the reviewer is great. Please add in to the text that the distribution of sample across UK is very representative - and add reference provided. The reader needs to know it is representative.

Response: Thank you for the comment. As suggested, we have added the following sentence to highlight the comparable aspect of our analytic sample to the original sample:

"This distribution closely mirrored that of the original sample, which was designed to be broadly representative of the total UK population at baseline through sampling techniques and weighting [39,86]*."*

15. The original wording is not included in the paper as far as I can see? if original wording is not being used, what is there needs further clarification. Need to include that the CM mother did not live with her parents at some point before age 17.

Response: We thank the reviewer for the comment. We have updated the text to include the original wording of the questions:

"Maternal care experience in childhood was derived from questions asking whether participants had spent any time living away from both parents before age 17. If yes, a follow-up question was made to participants to indicate where they mainly lived before age 17, including children's home, foster parents, boarding school, living with relatives, prison/young offenders institute/Borstal, or some other place. Mothers who had lived in a care placement, including foster care and children's home, were classified as having care experience"

16. I have amended text to be the EXACT wording; Prior maternal mental health: Scores from the modified Rutter Malaise Scale [41,42] were used to indicate the prior maternal mental health at 9 months postpartum. This scale includes nine binary items (1=yes, 0=no) about an individual's experience of the following symptoms: (1) 'feel tired most of the time', (2) 'often feel miserable or depressed', (3) 'often get worried about things', (4) 'often get into a violent rage', (5) 'often suddenly become scared for no good reason', (6) 'easily upset or irritated', (7) 'constantly keyed up or jittery', (8) 'every little thing gets on nerves and wears you out', (9) 'heart often races like mad' ....

Response: Thank you for the comment. We have updated the text as the reviewer suggested in the following manner:

"Prior maternal mental health: Scores from the modified Rutter Malaise Scale [41,42] were used to indicate the prior maternal mental health at 9 months postpartum. This scale includes nine binary items (1=yes, 0=no) about an individual experience of the following symptoms: (a) 'feel tired most of the time', (b) 'often feel miserable or depressed', (c) 'often get worried about things', (d) 'often get into a violent rage', (e) 'often suddenly become scared for no good reason', (f) 'easily upset or irritated', (g) 'constantly keyed up or jittery', (h) 'every little thing gets on your nerves and wear you out', (i) 'heart often races like mad"

24. Suggest including the level of missingness in your dataset for clarity for the reader. Include text in capitals - or something similar. "10 imputed datasets were generated based on Rubin's Rules [85] to estimate the uncertainty introduced by missing data, given the OVERALL LEVEL OF 10% missingness in our data."

Response: Thank you for the comment. As suggested by the reviewer, we have updated the text in the following manner:

"10 imputed datasets were generated based on Rubin's Rules [85] to estimate the uncertainty introduced by missing data, given the overall level of 10% missingness in our data."

Unrequested changes

On page 7, we have revised the text in the following manner to better reflect the sample:

"Of those care-experienced mothers, 60% (N=92) had experienced family-based care and 40% (N=64) had experienced group-based care."
